# Self-repair protects microtubules from their destruction by molecular motors

**DOI:** 10.1038/s41563-020-00905-0

**Published:** 2021-01-21

**Authors:** Sarah Triclin, Daisuke Inoue, Jérémie Gaillard, Zaw Min Htet, Morgan E. DeSantis, Didier Portran, Emmanuel Derivery, Charlotte Aumeier, Laura Schaedel, Karin John, Christophe Leterrier, Samara L. Reck-Peterson, Laurent Blanchoin, Manuel Théry

**Affiliations:** 1 University of Grenoble-Alpes, CEA, CNRS, INRA, Interdisciplinary Research Institute of Grenoble, Laboratoire de Physiologie Cellulaire & Végétale, CytoMorpho Lab, 38054 Grenoble, France; 2 Department of Human Science, Faculty of Design, Kyushu University, 815-8540, Fukuoka, Japan; 3 Deptartment of Cellular and Molecular Medicine, and Cell and Developmental Biology Section, Division of Biological Sciences, University of California San Diego, 9500 Gilman Drive, La Jolla, CA 92093, USA; 4 Howard Hughes Medical Institute, Chevy Chase MD, USA; 5 CRBM - CNRS UMR 5237, Route de Mende, 34293 Montpellier Cedex 05, FRANCE; 6 MRC Laboratory of Molecular Biology, Francis Crick Avenue, Cambridge Biomedical Campus, Cambridge CB2 0QH, UK; 7 University of Geneva Department of Biochemistry, Science II 30, Quai Ernest-Ansermet 1205 Genève, Switzerland; 8 University of Grenoble-Alpes, CNRS, Laboratoire Interdisciplinaire de Physique, 38000 Grenoble, France; 9 University of Aix Marseille Université, CNRS, INP UMR7051, Marseille, France; 10 University of Paris, INSERM, CEA, Institut de Recherche Saint Louis, U976, HIPI, CytoMorpho Lab, 75010 Paris, France

## Abstract

Microtubule instability stems from the low energy of tubulin dimer interactions, which sets the growing polymer close to its disassembly conditions. Molecular motors use ATP hydrolysis to produce mechanical work and move on microtubules. This raises the possibility that the mechanical work produced by walking motors can break dimer interactions and trigger microtubule disassembly. We tested this hypothesis by studying the interplay between microtubules and moving molecular motors in vitro. Our results show that molecular motors can remove tubulin dimers from the lattice and rapidly destroy microtubules. We also found that dimer removal by motors was compensated for the insertion of free tubulin dimers into the microtubule lattice. This self-repair mechanism allows microtubules to survive the damage induced by molecular motors as they move along their tracks. Our study reveals the existence of coupling between the motion of molecular motors and the renewal of the microtubule lattice.

Molecular motors can alter microtubule growth and shrinkage ^1^. Kinesins impact microtubule growth by regulating the dynamics of dimer addition or the recruitment of macro-molecular complexes at microtubule ends ^1,2^. Dyneins, the other family of microtubule-based motors, can capture microtubule plus ends and induce slow microtubule depolymerisation ^3^. The kinesin-13 and kinesin-8 families actively depolymerise microtubules by removing dimers at microtubule ends ^4,5^. These motors travel along the shaft and modulate microtubule dynamics as they reach microtubule ends, but have not been shown to have any impact on the microtubule shaft. However, thermal forces are sufficient to promote the removal of tubulin dimers from the lattice ^6^ and motors locked into a non-moving state are capable of expanding the microtubule lattice ^7,8^, suggesting that the mechanical work produced by moving motors could impact the stability and dynamics of microtubule shaft.

In vitro studies showed that microtubules can loose protofilaments or dimers as they glide on layers of motors ^9,10^. However, in those experiments, as in most in vitro studies of the mechanical forces produced by molecular motors, microtubules were stabilised in order to block microtubule depolymerisation ^11^. These conditions do not reflect the physiological conditions in which GTP-tubulin is rapidly hydrolysed to GDP-tubulin following tubulin dimer incorporation at the growing microtubule end. Because the GDP-tubulin lattice is less stable than that with GTP-tubulin, lattice dismantling may be more frequent than suggested by early experiments. Here we set out to study the impact of mechanical forces produced by kinesin and dynein motors on microtubules under conditions where the lattice would be made up of GDP-tubulin. We started by using microtubule “gliding assays”, where motors are attached to the glass surface of a small flow cell. Upon the addition of microtubules and ATP the forces produced by active motors result in microtubule gliding across the motor-coated surface.

## Molecular motors can destroy non-stabilized microtubules

**Figure 1:**
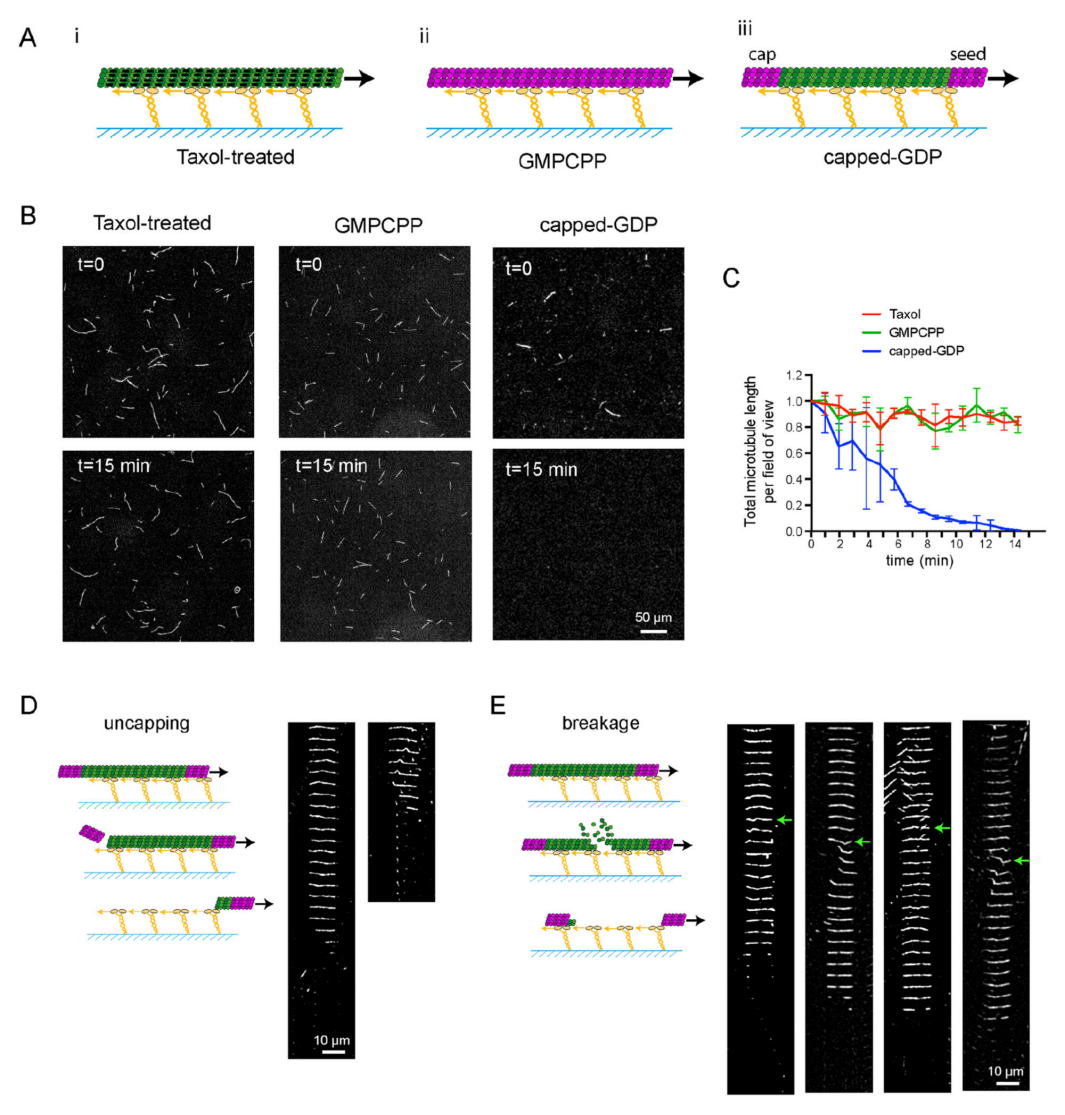
Gliding assays destroy non-stabilized microtubules A - Schematic representation of the different types of microtubules used in gliding assays. Taxol-treated (purple) (i) and GMPCPP-microtubules (magenta dimers) (ii) are stabilized along their length. Capped-GDP microtubules (iii) are stabilized at their ends (magenta dimers) but the central part (green dimers) is not. Molecular motors (yellow) are attached to the cover glass (blue), and propel microtubules along the glass surface in the presence of ATP. B - Images of gliding microtubules when motors are activated by the addition of ATP (t=0, top row) and 15 minutes later (t=15 min, bottom row) in the three conditions described in A. Scale bar: 50 μm. C - Quantification of microtubule length variations in the experiments shown in B. The microtubule lengths were measured for all microtubules in the 600-μm-wide fields every minute during 15 minutes. Values were normalized with respect to the initial length (Number of independent experiments N=2, Taxol-treated microtubules n=55; GMPCPP-microtubules n=42; capped-GDP microtubules n=42). Error bars represent standard deviation. D - Schematic representation of microtubule disassembly induced by the loss of the cap. Two examples of microtubules that disassembled from an end during the gliding assay are shown. Images are vertical montages from a movie where images were acquired every 5 s. Scale bar: 10 μm. E - Schematic representation of microtubule disassembly induced by breakage of the central region of the microtubule. Four examples of microtubules that break and disassemble during the gliding assay are shown. Images are vertical montages from a movie where images acquired every 5 sec. Green arrows point to bending events that precede the weakening and eventual breakage of the microtubule. Scale bar: 10 μm.

We first performed gliding assays with the plus-end-directed human kinesin-1 on microtubules stabilized either with taxol (Figure 1A-i) or by assembling microtubules with GMPCPP-bound tubulin, which traps tubulin in a state that mimics stable GTP-bound tubulin (Figure A-ii). The total amount of microtubules did not change during the time-course of the assay in either condition (Figure 1B, 1C, Supplementary Movie 1). Next, to study the response of GDP-lattice microtubules, we capped microtubules on both ends with a short microtubule section consisting of GMPCPP-tubulin, which protects microtubules from depolymerising from their ends (Figure 1A-iii, see Methods). These capped-GDP microtubules started depolymerizing within minutes of initiating the gliding assay (Figure 1B, Supplementary Movie 2). Fifteen minutes later, no microtubules were detectable on the slide (Figure 1C). The destruction of microtubules in contact with surface-bound motors appeared to depend on the ATP concentration (Supplementary Figure 1A) and could not be observed in the absence of gliding, as previously reported ^7^.

To investigate the mechanism leading to the disassembly of GDP-microtubules, we tracked individual microtubules gliding on kinesin-1, which moves towards the plus ends of microtubules. The majority of the microtubules appeared to depolymerize from their plus end (Figure 1D), consistent with a previous report suggesting that kinesin-1 can trigger the loss of tubulin dimers at the end of stabilized microtubules ^9^. In addition, 20% of microtubules broke within the GDP lattice (Figure 1E, Supplementary Movie 3). In some cases, breakage events were observed when microtubules encountered other microtubules or when microtubule buckling was observed (Figure 1E, arrows), suggesting that in our assays multiple mechanisms that induce breakage could be in play.

**Figure 2:**
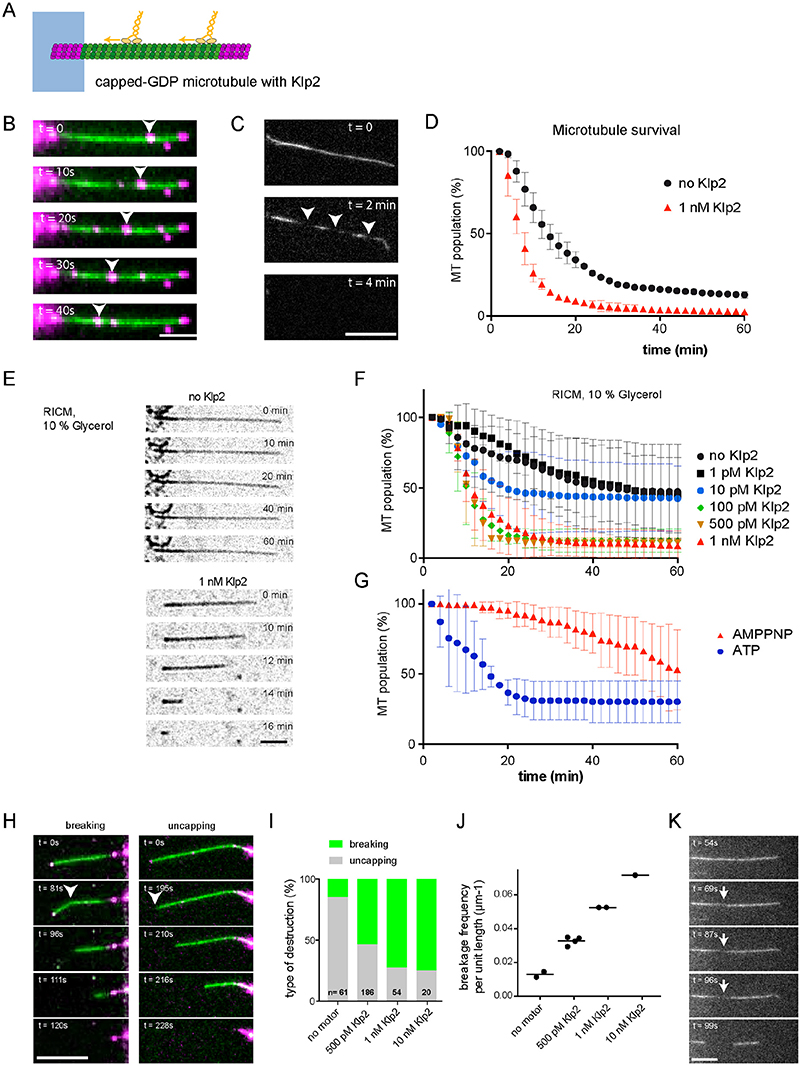
Motility assays destroy non-stabilized microtubules A - Schematic representation of the motility assay. The microtubule seed (magenta) is attached to the micropattern (blue), while the shaft (green) and the cap (magenta) are free. Motors (yellow) walk along the microtubules in the presence of ATP. B - Sequence showing labelled-Klp2 (magenta) walking toward minus-ends. Images were taken every 10 sec. The arrow heads point at a cluster of moving motors. Note that the seed and the cap are also visible in the green channel. Scale bar: 10 μm. C - Sequence showing the formation of breaks (arrow heads) in the microtubule shaft prior to disassembly. Motors were not labelled, and images were taken every 2 min. Scale bar: 10 μm. D - Survival curve of microtubules in the absence (blue) or presence (red) of motors. We measured the duration between the addition of motors and the disappearance of microtubules (Number of independent experiments N=2, control n=588, Klp2 n=1130. Pairwaise testing (non-parametric Wilcoxon Mann-Whitney (Wilcoxon rank sum test), two-tailed) of the measured lifetimes gives p-values < 0:0001. Error bars represent standard deviation. E - Sequence showing capped-GDP microtubule under RICM in the absence (Top) or presence (Bottom) of 1 nM Klp2. Images were taken every 2 min. Scale bar= 5μm F - Survival curve of microtubules in the absence (black circle) or presence of motors at 1 pM to 1 nM Klp2. Data were acquired in the conditions described in E. Number of independent experiments: no Klp2, N=9, n=371; 1 pM Klp2, N=2, n=120; 10 pM Klp2, N=2, n=136; 100 pM Klp2, N=4, n=176; 500 pM Klp2, N=2, n=81; 1 nM Klp2, N=5, n=317. Statistical test: see Supplementary figure 2 and Supplementary tables 2 to 9. Error bars represent standard deviation. G – Survival curve of unlabelled microtubules visualised with RICM in presence of 1 nM Klp2 in presence of ATP (blue curve) or AMPPNP (red curve). (Number of independent experiments: with ATP, N=2, n=142; with AMPPNP, N=2, n=89). Statistical test: see Supplementary tables 6 to 9. Error bars represent standard deviation. H - Sequence showing examples of microtubule breakage (left, arrow head) or cap loss followed by depolymerization (right, arrow head). The microtubule lattice in green and the GMPCPP cap in magenta. Scale bar: 10 μm. I – Bar graph showing the relative proportion of microtubule disassembly after the loss of their cap (grey) or a breakage in their central part (green) depending on the concentration of Klp2. J - Graph showing the spatial frequency of microtubule breakage as a function of the concentration of Klp2. Number of independent experiments and number of microtubules: no Klp2, N=2, n=61; 0,5 pM Klp2, N=4, n=186; 1 nM Klp2, N=2, n=54; 10 nM Klp2, N=1, n=20. K - Sequence showing the local loss of tubulin dimers in the lattice (arrows) prior to lattice breakage. Scale bar: 10 μm.

To distinguish between breaking mechanisms we next performed “motility assays” where microtubule-based motility was studied in the opposite geometry, with capped GDP-microtubules attached to the coverslip surface and motors walking along them ^12^. Typically, in these assays the microtubules are attached to the surface along their entire length. To attempt to limit these surface interactions (which could interfere with microtubule damage), we used micropatterning to attach microtubule seeds to the surface, leaving the rest of the capped–GDP microtubule unattached to the non-adhesive surface coating ^13^ (Figure 2A).

We first tested the impact of Klp2, a *Schizosaccharomyces pombe* member of the minus-end-directed kinesin-14 family ^14^, on the stability of capped-GDP microtubules. Klp2 motors fluorescently labelled with ATTO488 moved towards the microtubule minus-end at 120 nm/s, similar to previous reports (Figure 2B) ^15^. To limit photo-induced damage we next used non-labelled Klp2 and followed microtubules breaking (Figure 2C, Supplementary Movie 4) and disappearing over the course of an hour. In the absence of kinesins about 86% of microtubules disappeared spontaneously with a lifetime of about 12.3 ± 0.1 minutes (Figure 2D, see Supplementary Methods and Supplementary Table 1 for statistical test and life time estimation). In the presence of 1 nM Klp2, 96% of microtubules disappeared with a lifetime of about 5.3 ± 0.1 min (Figure 2D and Supplementary Table 1). Note that the mean lengths of microtubules for both conditions were not significantly different (15±0.7 μm and 13.2±0.6 μm (average ± sem) for the control and at 1 nM Klp2, respectively) so the duration for disassembly were similar in both conditions.

The effect of Klp2 on microtubule lifetime was partially hindered by the fact that a significant proportion of capped microtubules disappeared spontaneously; an effect that stems from structural defects in the lattice ^6^ and fluorescence photodamage ^16,17^. In order to further reduce the photo-toxicity effects and increase the intrinsic stability of microtubules, we combined the use of reflection interference contrast microscopy (RICM) to visualize label-free microtubules ^18^ with the use of 10% glycerol in the reaction mixture (Figure 2E, and Supplementary Figure 1C). In these conditions, only 69% of microtubules disappeared during the hour that lasted the experiment, and their lifetime was extended to 20 ± 2 minutes (Figure 2F). In the presence of 1 nM Klp2, this proportion increased to 86% and their average lifetime was reduced to 8.7 ± 0.2 minutes. Similar effects were observed with 500 pM and 100 pM of Klp2. They were diminished with 10 pM of Klp2 and undetectable at 1pM (Figure 2F). In the presence of AMPPNP, a non-hydrolysable form of ATP, motors bind to microtubules, but do not move ^19^. When we assayed microtubule breakage in the presence of Klp2 and AMPPMP in our experiments we did not observe microtubule breakage ^7^ (Figure 2G). The visualisation of the microtubule shaft and cap with distinct fluorescent labels allowed us to distinguish that the ability of Klp2 to induce shaft breakage, instead of cap removal (Figure 2H, Supplementary Movie 5). Several microtubules were imaged in a row to quantify the relative proportions of breakage and uncapping. Interestingly, the proportion of shaft breakage increased with increasing motor concentration (Figure 2I). At 10 nM Klp2 we measured an average of one breaking event per 14 microns of microtubule (Figure 2J), corresponding in some cases to multiple breaks per microtubule (Supplementary Movie 4). Together our data thus far show that kinesin motors damage microtubules as they move along them in a manner that leads to microtubule breakage.

## Single dimers of dynein can destroy non-stabilized microtubules

**Figure 3:**
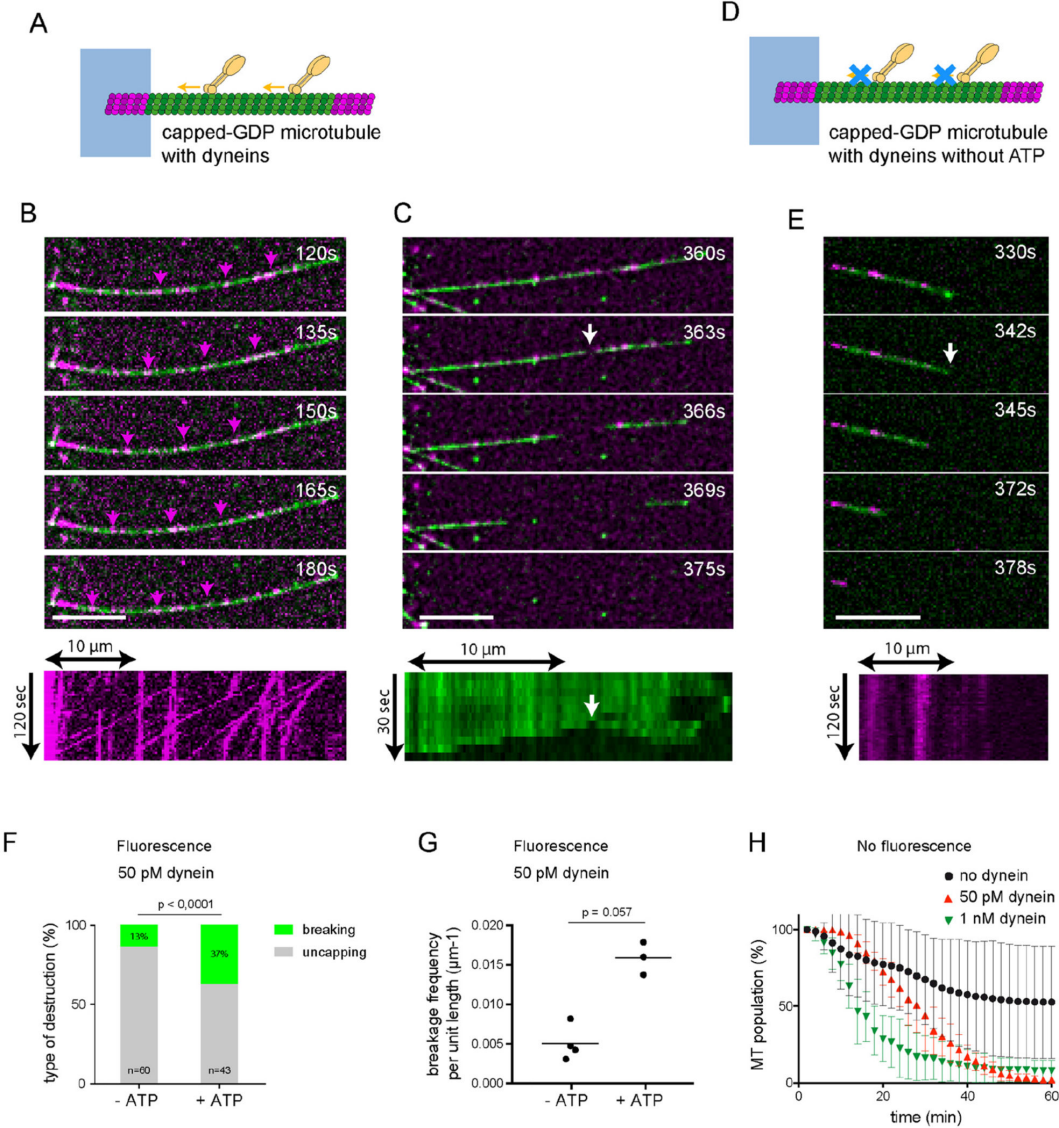
Single dyneins can damage the microtubule lattice. A - Schematic representation of the motility assay with dyneins. The microtubule seed (magenta) is attached to the micropattern (blue), while the shaft (green) and the cap (magenta) are free. Molecular motors (dyneins, yellow) walk along the microtubules toward microtubule minus-end in the presence of ATP. B - Image sequence showing single dyneins (magenta, 50 pM) walking along a micropatterned capped microtubule (green). The magenta arrows track moving dyneins. Images were taken every 3 sec. Scale bar: 10 μm. The kymograph shows the traces of single dyneins (50 pM) moving toward microtubule minus-end on the micropattern. C- Image sequence illustrating lattice breakage (white arrow). Scale bar: 10 μm. The kymograph below shows the microtubule shaft and its disassembly toward both ends from the breaking point. D - Same schematic representation than in A. Molecular motors (yellow) do not walk along the microtubules in the absence of ATP. E - Image sequence showing single dyneins (magenta) binding to a micropatterned capped microtubule (green). The white arrow points at the loss of the GMPCPP cap that is followed by microtubule disassembly. The kymograph below shows the traces of single dyneins that are not moving on the microtubule. Scale bar: 10 μm. F - Bar graph showing the relative proportion of microtubule disassembly after the loss of their cap (grey) or a breakage in their central part (green) in response to dynein (50 pM) depending on the presence or absence of ATP. With ATP, N=3 independent experiments and 43 microtubules in total, without ATP, N=4 independent experiments and 60 microtubules. The p value was measured with a Fisher exact test. G - Graph showing the spatial frequency of microtubule breakage in response to dynein (50 pM) depending on the presence or absence of ATP. With ATP, N=3 independent experiments for a total length of 1298 μm. Without ATP, N=4 independent experiments for a total length of 1750 μm. The p value was measured with a Fisher exact test. H - Survival curve of microtubules in the absence (black circle) or presence of dyneins at 50 pM (red triangle) or 1 nM (green triangle). Dyneins were not labelled and capped-microtubules were not labelled. Data were acquired with RICM, ie in the absence of fluorescence, and in the presence of glycerol. Number of independent experiments: no Dynein, N=9, n=371; 50 pM Dynein, N=2, n=211; 1 nM Dynein, N=2, n=152. Statistical test: see Supplementary figure 2. Error bars represent standard deviation.

Klp2 is a non-processive motor ^20^ so the long walking distances we observed suggested they formed aggregates in our experimental conditions. Clusters of motors likely represent the mechanism by which they share the load as they transport vesicles along microtubules in cells and therefore constitute physiologically relevant conditions ^21^. However, we also wanted to test whether single motors could also destroy the microtubule lattice. To do this, we used cytoplasmic dyneins that were purified from *Saccharomyces cerevisiae* ^22^ since they exist as single dimeric molecules when used at 50 pM ^23^ (Supplementary Figure 3). We performed motility assays with capped GDP-microtubules (Figure 3A) and tracked single fluorescently-labelled dyneins as they moved along microtubules (Figure 3B, Supplementary Movie 6). In these conditions, we observed clear examples of lattice breakage followed by microtubule disassembly (Figure 3C, Supplementary Movie 7). Of note, in the absence of ATP, dyneins did not move and microtubule disassembly was generally induced by the spontaneous loss of the GMPCPP cap (Figure 3D, E). Indeed, lattice breaking events in response to single dyneins (50 pM) were twice as frequent in the absence of ATP compared to the presence of ATP (15% n=13 versus 34% n=36) (Figure 3F), and the spatial frequency of lattice breakage increased from 0.4 to 1.4 per hundred micrometer of microtubule (Figure 3G).

With RICM we could further test the impact of non-fluorescently labelled dyneins on non-labelled microtubules. Similar to kinesins, dyneins forced microtubule destruction at 1 nM: 96% of microtubules disappeared in the hour following addition of motors and their lifetime was reduced from 20 to 12.4 ± 0.3 min (Figure 3H). In the presence of single dyneins at 50 pM, the effect was reduced (the average lifetime, 21.6 ± 0.6 min, was not significatively different from control), but clearly detectable, as most microtubules were destabilized after one hour whereas 40% were still present in the absence of motor (Figure 3H and Supplementary Figure 2D and Table S10 to S14 for statistical analysis). Altogether these results show that single motors impact microtubule lattice stability. This is surprising since single motors in this assay are not supporting any load, and therefore do not develop counteracting forces on the microtubule they walk on. However, ATP hydrolysis during the motor cycle releases more energy than is required to displace motors at the observed speed ^24^. One possibility is that some of this energy is transferred to the microtubule lattice and breaks dimer interactions, facilitating their detachment from the protofilaments.

## Free tubulin protects microtubules from motor-induced destruction

Interestingly, in both motility and gliding assays, most breaking events were preceded by a local reduction of microtubule fluorescence, which we hypothesized was due to the loss of tubulin dimers (Figure 1E, Figure 2K arrow). These observations raised the possibility that free tubulin dimers in solution could compensate for this loss by incorporating into the damaged region and protect the microtubule from breakage ^25,26^.

**Figure 4:**
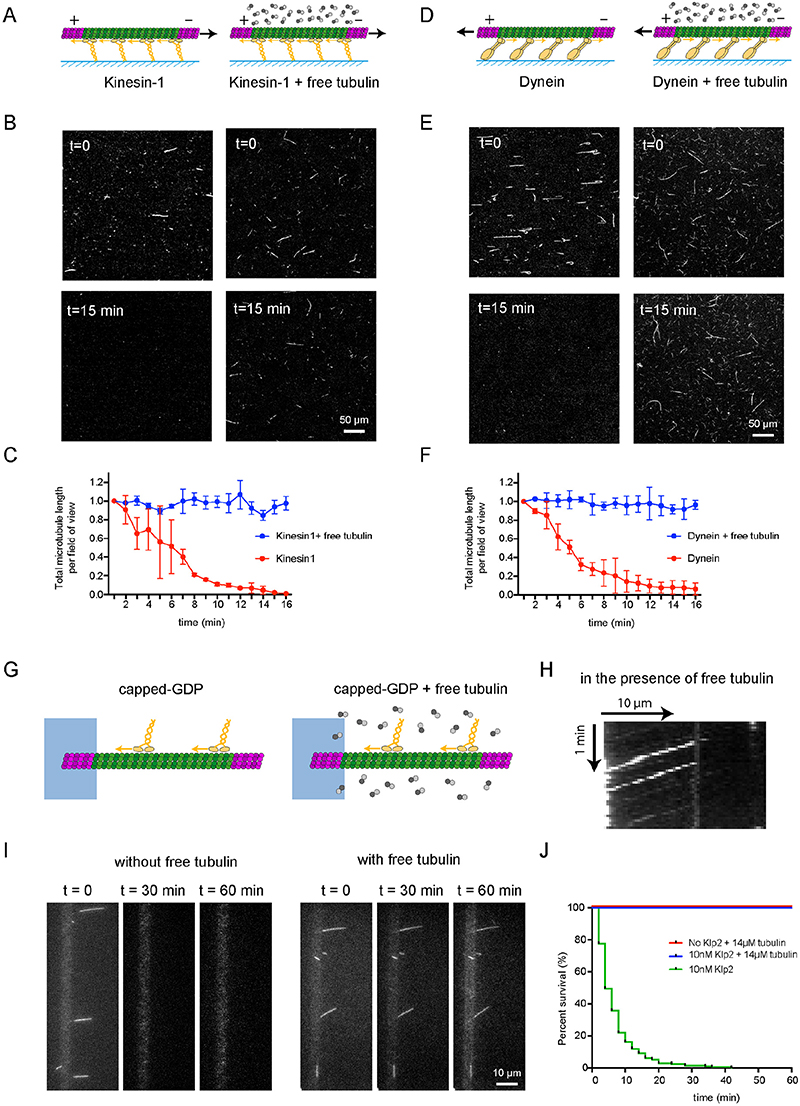
Free tubulin dimers prevent microtubule destruction by kinesin and dynein A - Schematic representation of the gliding assay of capped-GDP microtubules on kinesin-1 in the absence (left) or presence (right) of free non-labelled tubulin dimers. B - Images of gliding microtubules when motors are activated by the addition of ATP (t=0, top row) and 15 minutes later (t=15 min, bottom row) in the two conditions described in A. Scale bar: 50 μm. C - Quantification of microtubule length variations in the experiments shown in B. The microtubule lengths were measured for all microtubules in the 600-μm-wide fields every minute during 15 minutes. Values were normalized with respect to the initial length. Data in the absence of free tubulin dimers are shown in red and correspond to the data shown in figure 1C, those in the presence of 14μM of free dimers are shown in blue. Values were normalized with respect to the initial intensity (Number of independent experiments N=2, without free tubulin n=42, with free tubulin n=74). Scale bar: 50 μm. Error bars represent standard deviation. D,E,F – Same as A, B and C in the presence of yeast dynein instead of kinesin-1. (N=2, without free tubulin n=24, with free tubulin n=12). Error bars represent standard deviation. G - Schematic representation of the motility assay in the absence (left) and presence (right) of free non-labelled tubulin dimers. H - Kymograph showing the position of fluorescent-Klp2 along a microtubule over time. The presence of free dimer did not interfere with the ability of the motor to walk towards microtubule minus ends. I - Image sequences show microtubules after the addition of Klp2 motors in the absence (left) or presence (right) of 14μM of free tubulin dimers. Images were taken every 2 min. Scale bar: 10 μm. J - Survival curve of microtubules in the presence of molecular motors and absence of free tubulin (green), in the presence of molecular motors and 14μM of free tubulin dimers (blue) or in the absence of motors and presence of 14μM of free tubulin dimers (red). Data where acquired in the conditions described in I. (N=1; No motor, n=108; 10nM Klp2 with free tubulin, n=70; 10nM Klp2 without free tubulin, n=212).

To test this hypothesis, gliding assays were performed in the presence of free tubulin dimers (Figure 4A). Free tubulin dimers were unlabelled to allow microtubules to be monitored without background fluorescence. Strikingly, capped-microtubules glided on kinesin-1 in the presence of tubulin dimers without any visible destruction Figure 4B, C, movie S8). Microtubule gliding speed were ~ 500 nm/s in the presence or absence of free dimers (500±75 nm/s and 480±70 nm/s, respectively), showing that free dimers do not interfere with the motor-driven gliding speed of microtubules. The same observation was made in the presence of dynein. Gliding on dyneins in the absence of free dimers led to rapid microtubule disassembly, which could be prevented by the addition of free tubulin dimers (Figure 4D, E, F, movie S9). Similarly, the addition of free dimers in motility assays (Figure 4G) did not interfere with motor movement (Figure 4H), but fully protected microtubules from motor-induced breakage (Figure 4I, J, movie S10).

## Molecular motors catalyse the self-repair of microtubule lattice

**Figure 5:**
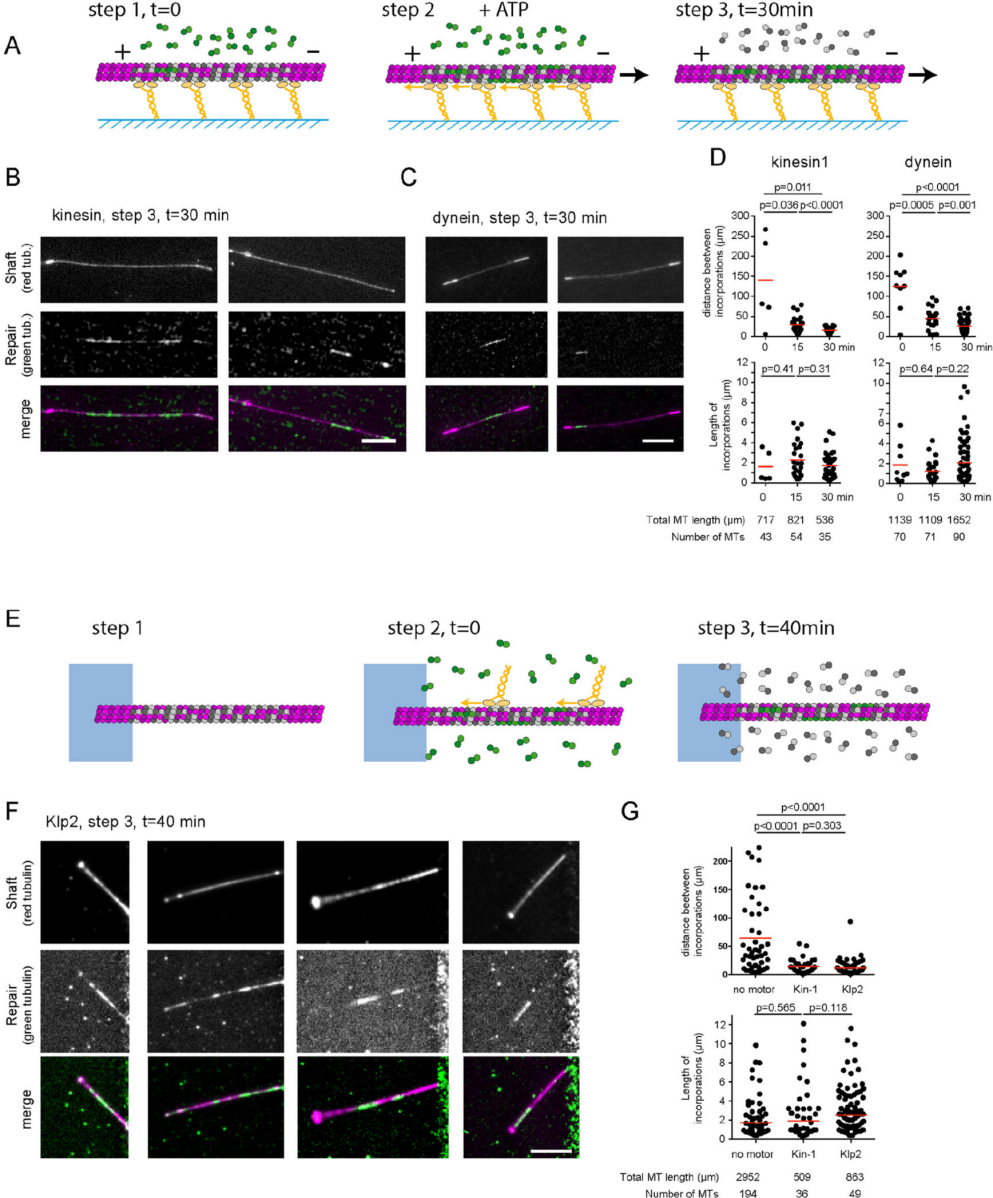
Molecular motors enhance tubulin exchange in the microtubule lattice. A - Schematic representation of the three sequences during gliding assays of capped-GDP microtubules in the presence of 14μM of free fluorescent tubulin. All experiments lasted 30 minutes. Only the duration of the gliding step was varied. Step1 corresponds to the loading of capped-GDP microtubules and free green tubulin dimers on the layer of motors. Step 2 corresponds to the initiation of gliding by the addition of ATP. In the 0-minute condition, ie without gliding, step 2 was skipped and ATP was not added. In the 15-minute condition, step 2 was initiated 15 minutes after step 1. In the 30-minute condition, step 2 immediately followed step 1. In all cases, fluorescent tubulin were removed after 30 minutes to measure the incorporation of free tubulin. B, C – Images show the microtubule shaft (low intensity) with associated cap and seed (high intensity) (top row), the incorporation of free tubulin (middle row), and the overlay of the two signals (bottom row) at the end of the gliding assay (step 3). In these examples, microtubules glided on kinesin-1 (B) or dynein (C) for 30 minutes. Scale bar: 10 μm. D – Quantifications of the spatial frequency (top) and size (bottom) of the incorporation sites upon gliding on kinesin-1 (left) or dynein (right) depending on the duration of the gliding step. Data were acquired during two independent experiments. Each experiment allowed the measurement of a single value of incorporation frequency over several microtubules. Red bars show geometrical means with 95% confidence interval. P-values were obtained using a non-parametric Mann-Whitney test. The table indicates the number and total length of microtubules that were measured in each condition. E - Schematic representation of the three sequences during motility assays on capped-GDP microtubules in the presence of 14μM of fluorescent tubulin. Step 1 is the growth and capping of microtubules. Step 2 corresponds to the addition of motors and fluorescent tubulin. Step 3 corresponds to the removal of motors and replacement of fluorescent tubulin by non-labelled tubulin 40 minutes later. F - Images show the microtubule shaft (top row), the incorporation of free dimers (middle row), and the overlay of the two signals (bottom row) at the end of the motility assay. Scale bar: 10 μm. G - Quantification of the spatial frequency (top) and size (bottom) of the incorporation sites following the motility of kinesin-1 (10 nM) or Klp2 (10 nM). Data were acquired during four independent experiments for the control conditions without motors, a single experiment with kinesin-1 and two independent experiments with Klp2. Each experiment allowed the measurement of a single value of incorporation frequency over several microtubules. Red bars show geometrical means with 95% confidence interval. P-values were obtained using a non-parametric Mann-Whitney test. The table indicates the number and total length of microtubules that were measured in each condition.

To directly visualize microtubule repair in response to motor-induced destruction, we performed a two-color assay in which polymerized tubulin and free tubulin dimers were labelled with distinct fluorophores. Free fluorescent dimers were then washed away and replaced by non-labelled free tubulin in order to protect microtubules from spontaneous breakage and disassembly in the absence of free tubulin ^6^. This allowed us to visualize tubulin incorporation at damaged sites ^26^ (Supplementary Figure S4). In a kinesin-1 gliding assay in the presence of free green-tubulin dimers (Figure 5A), red-fluorescent capped-GDP microtubules displayed micrometer-long green stretches along their shaft 30 minutes after the initiation of the gliding assay (Figure 5B). These two colored microtubules could also be seen moving along the kinesin-1 surface (movie S11). Similar two-colored microtubules were observed in dynein microtubule gliding assays performed in the same manner (Figure 5C). By varying the duration of the gliding step during the 30-minute assay (step 2 in Figure 5A) we found that the size of the repaired sites remained constant, but that their frequency along the shaft increased regularly (Figure 5D). This suggests that motors continuously generate new sites of damage. Note that the presence of repaired sites in microtubules which did not glide because of the absence of ATP during the 30 minutes of the assay (“time 0” in figure 5D, examples in Supplementary Figure S5) was consistent with our recent observation that microtubule lattice has an intrinsic turnover rate ^6^. In gliding microtubules, the increased frequency of repair sites corresponded to the addition of the intrinsic and the motor-induced contributions. The spatial frequency of the repaired sites was lower in the dynein gliding assay compared to the kinesin assay, perhaps due to differences in the force production and velocity of these two motors. Yeast dynein moves about 10-fold slower than kinesin-1 and also has a slightly lower stall force ^27^.

The integration of free tubulin dimers into the microtubule shaft could also be visualized in motility assays using a similar experimental strategy (Figure 5E). Clear stretches of green tubulin dimers along the shaft of red microtubules were present on 80% (n=85) of microtubules after kinesin-1 or Klp2 moved along them (Figure 5F, movie S12). The frequency of the repaired sites was similar to those measured in gliding assays, but the size of the repaired sites was larger (Figure 5G). In some occasions, the intensity of red fluorescence appeared correlated to the decrease of green fluorescence but it was not always the case because of the low labelling frequency in the green lattice (Supplementary Figure S6). Together these results show that walking motors can break the microtubule lattice, catalysing the incorporation of free tubulin dimers, which heal the lattice and protect it from disassembly. From this data, we infer that components of the microtubules that are supporting motor trafficking are continuously renewed.

At this point, we can only speculate about the detailed molecular mechanism of lattice destruction and repair. The increasing amount of lattice repair sites over time (Fig. 5D, top) supports the idea that motors do not only enhance the exchange of dimers at pre-existing defects such as dislocations ^6^ but also generate additional sites of tubulin removal and turnover. By contrast, the constant length of repair sites (Fig. 5D, bottom) and their micrometer range (Fig. 5D and 5G) suggest that the propagation is determined by a collective process of dimer loss and replacement until the defect is annihilated and the exchange dynamics stops rather than the result of motor activity.

## Conclusion

Whether motor-induced destruction and repair of microtubules happens in cells remains to be established. The localisation of GTP-tubulin ^28^ or microtubule-associated proteins mediating microtubule repair such as CLIP-170 ^29^ and CLASP ^30^, can serve as a proxy for the identification of repair sites in living cells. These sites are numerous along microtubules submitted to high conformational stress ^29^, but also along straight microtubules in axons ^31^, where motor-based trafficking is particularly intense, supporting our hypothesis that motors are involved in this process. Importantly, the incorporation of new GTP-tubulin dimers generates rescue sites along microtubules, increasing their lifespan ^29,32,33^. This raises the possibility that dynein and kinesin motors may trigger the selective stabilization of the microtubules they are walking on, which could be instrumental in the definition of preferential tracks in microtubule networks and the establishment of cell polarity.

## Methods

### Microtubule gliding assay

In vitro gliding assays were performed using flow chamber with dimensions of 2 × 10 × 0.07 mm (W × L × H), which were assembled from 10 × 10 mm and 24 × 30 mm cover glasses (Thermo Scientific) with double-sided tape as the spacer. Anti-GFP antibody (Invitrogen) at 0.2 mg/mL (5 μL) was applied to the flow chamber for gliding assays with kinesins. The flow chamber was washed with 5 μL of 1% w/v BSA in HKEM (10 mM HEPES pH=7.2, 50 mM KCl, 1 mM EGTA, 5 mM MgCl_2_). Next 5 μL of 200 nM GFP-tagged kinesin-1 or 3 μL of 10 nM yeast dynein in TicTac buffer (10 mM HEPES, 16 mM PIPES (pH 6.8), 50 mM KCl, 5 mM MgCl_2_, 1 mM EGTA, 20 mM DTT, 3 mg/ml glucose, 20 μg/ml catalase, 100 μg/ml glucose oxidase, and 0.3% BSA) was introduced for 3 min and washed with 10 μL wash buffer. 10 μL of microtubules in HKEM was then introduced and incubated for 1 min, followed by extensive washing with TicTac buffer. Finally, 10 μL of ATP (2.7 mM final, Sigma Aldrich, A3377) in TicTac buffer supplemented with 0.2% Methyl cellulose was added into the flow chamber prior to imaging. In the experiment with taxol-stabilized microtubules, washing buffers and ATP buffers contained 10 μM taxol. In the experiments in the presence of free tubulin, 14 μM of unlabelled tubulin was added to the ATP buffer.

### Microtubule motility assay

Micropatterning of glass slides and fabrication of the microfluidic circuitry are described in the Supplementary methods section.

Microtubule seeds were prepared at 10 μM tubulin concentration (20% ATTO647-labelled and 80% biotinylated tubulin) in BRB80 (80mM PIPES pH=6.8, 1mM MgCl_2_, 1mM EGTA) supplemented with 0.5 mM GMP-CPP at 37°C for 1 h. The seeds were incubated with 1 μM Taxotere (Sigma) at room temperature for 30 min and were then sedimented by centrifugation at 30°C for 15min at 100,000 x g and resuspended in BRB80 supplemented with 0.5 mM GMP-CPP and 1 μM Taxotere. Microtubule seeds were stored in liquid nitrogen and quickly warmed to 37°C before use.

The Poly-DiMethyl-Siloxane (PDMS) chip was placed on a micropatterned cover glass and fixed on the microscope stage. The chip was perfused with neutravidin (50 μg/ml in HKEM; Pierce), then washed with HKEM supplemented with 0.2% BSA and passivated for 1 min with PLL-g-PEG (Pll 20K-G35-PEG2K, Jenkem Technology) at 0.1 mg/ml in 10 mM Na-Hepes (pH 7.4), and washed again with HKEM plus 0.2% BSA. Microtubule seeds were flowed into the chamber at high flow rates perpendicularly to the micropatterned lines to ensure proper orientation of the seeds. Non-attached seeds were washed out immediately using HKEM supplemented with 0.2% BSA. Seeds were elongated with a mix containing 20 μM tubulin (5 or 20% labelled with a ATTO-488 or ATTO-565 fluorophore) in TicTac buffer (10 mM HEPES, 16 mM PIPES (pH 6.8), 50 mM KCl, 5 mM MgCl_2_, 1 mM EGTA, 20 mM DTT, 3 mg/ml glucose, 20 μg/ml catalase, 100 μg/ml glucose oxidase, 0.3% BSA and 0.25% methyl cellulose (1500 cp, Sigma)) for 20 min. GMPCPP caps were grown by supplementing the before-mentioned buffer with 0.5 mM GMPCPP (Jena Bioscience) instead of GTP and using 14 μM tubulin (100% labelled with a ATTO-488 or ATTO-565 fluorophore) for 15 min. To monitor microtubule survival in the absence of free tubulin, the same buffer as for seed elongation was used, but without free tubulin. To quantify the effect of molecular motors on microtubules survival following the capping step, the same buffer as for seeds elongation was used, but supplemented with molecular motors (at the appropriate concentration) and 2.66 mM ATP or AMP-PNP (Sigma Aldrich 10102547001).

To analyse non-fluorescently labelled microtubules using RICM (see below), seeds were prepared by polymerizing 10 μM of non-fluorescent tubulin, 20% of which was biotinylated, and attached to neutravidin-coated micropatterns. Grafted seeds were further elongated with 20 μM of unlabelled tubulin, and then capped by adding 10 μM of unlabelled tubulin with 30 μM GMP CPP. The capping medium was then replaced by the elongation buffer described above, but without tubulin and supplemented with 10% of glycerol to increase microtubule stability. Unlabelled kinesins or dyneins were added to this buffer at the concentrations reported in the main text.

### Visualization of free dimer incorporation

To visualize incorporation of free tubulin dimers into the microtubule shaft during gliding, the glass surface was passivated with 1% w/v BSA and 1% w/v pluronic F-127. Unlabelled SNAP-kinesin1-His was used instead of GFP-kinesin1-His. Green fluorescent dimers tend to attach to the layer of motors during the gliding assay, hindering the detection of the few dimers that may incorporate into the lattice. To limit this effect, the last coating step was performed with wash buffer containing 14 μM unlabelled tubulin. Capped-GDP microtubules were assembled in a single red colour, but with a lower proportion of fluorescent dimers (3%) in the central GDP part compared to stabilized ends (20% labelled tubulin). Fluorescence in the central part of the microtubule was required to visualize capped-microtubules and distinguish them from individual seeds. The gliding of these capped-microtubule was initiated with ATP buffer and recorded for 30 min. In the control experiment, ATP was not included in the reaction mixture. At the end of the gliding experiments, labelled tubulin was washed out and replaced by new ATP buffer with 7 μM unlabelled tubulin in order to visualize green dimer incorporation into the capped-microtubules without triggering microtubule destruction due to dimer washout.

To visualize incorporation of free tubulin dimers into the microtubule shaft during motility assays, capped-microtubules were exposed to 14 μM of tubulin (100% labelled, green or red fluorescent) in 10 mM HEPES, 16 mM PIPES (pH 6.8), 50 mM KCl, 5 mM MgCl_2_, 1 mM EGTA, 20 mM DTT, 3 mg/ml glucose, 20 μg/ml catalase, 100 μg/ml glucose oxidase, 0.3% BSA and 0.25% methyl cellulose (1500 cp, Sigma), in the absence or presence of motors, for 40 min before replacing it with elongation buffer in which fluorescent tubulin dimers were replaced by 10 μM unlabelled tubulin in order to protect microtubules from disassembly without perturbing the imaging process with background fluorescence.

### Imaging

Large-scale measurement of microtubule lifetime during gliding assays were performed by illuminating the sample with a SOLA Light engine (Lumencor) and visualised with an epifluorescence microscope (Eclipse Ti2, Nikon) using a S Plan Fluor 20X objective lens (Nikon). The microscope stage was kept at 37°C using a warm stage controller (LCI). Images and movies were captured using a CMOS camera (Hamamatsu) every 5 sec for 15 min.

Individual microtubule destruction or repair events during gliding and motility assays were visualized using an objective-based TIRF composed of a Nikon Eclipse Ti, an azimuthal ilas2 TIRF illuminator (Roper Scientific), a 60x NA 1.49 TIRF objective lens followed by a 1.5X magnification lens and an Evolve 512 camera (Photometrics). The microscope stage was kept at 37°C using a heated stage controller (LCI). Excitation was achieved using 491 and 565 nm or 642 nm lasers (Optical Insights). Time-lapse recording was performed using Metamorph software (version 7.7.5, Universal Imaging). Images were taken every second.

Large scale measurement of microtubule lifetime during motility assays were performed by acquiring images every 2 min for 1 hour. To detect microtubule breakage, images were taken every 3 sec until microtubule disappeared. To visualize free tubulin dimer incorporation into polymerized microtubules, images were taken every sec for 15 sec at the end of the assay so that images could be averaged to improve the signal to noise ratio.

Non-labelled microtubules and non-labelled motors were imaged with Reflection Interference Contrast Microscopy ^18^: the sample was illuminated with a SOLA Light engine (Lumencor) through a cube equipped with a monochromatic filter at 520 nm and a 50/50 dichroic mirror and a 60x NA 1.49 TIRF objective.

### Image processing and measurements

For gliding assays, microtubule lifetime was quantified by measuring the variation of the microtubule length over time by using the line tool of Adobe Photoshop CC2018. The quantification of the free tubulin incorporation along the microtubule shaft was done using a line scan in Image J after the following image treatment. We arbitrary selected three frames at different time points for the green and red channels (incorporated tubulin is green and the microtubule is red). Overlaid images were generated using an Adobe Photoshop original macro for each channel by cancelling the displacement of the microtubule during gliding. To generate the image overlay, the Photoshop layer mode “darken mode” was applied to each image in order to reduce background noise. Finally, the background of the overlaid image was subtracted by Image J (Rolling ball radius 10.0 pixels). The incorporation was defined as signal 1.5 times higher than background (see Supplementary Fig. S1).

For motility assays, movies were processed to improve the signal/noise ratio. Background was subtracted with a dedicated plug-in from imageJ, version 1.47n5). Microtubule lifetime was quantified by manually counting microtubule number at each frame, every 2 min for 1 hour. To quantify the incorporation of free tubulin dimers, line scans were used to measure fluorescence intensity along the microtubule. Incorporation was counted when a spot perfectly aligned with the microtubule lattice, followed microtubule lateral fluctuations and the fluorescence intensity showed a peak at least 2-fold higher than the background fluorescence.

### Statistical analysis.

Statistical analysis and chart design were performed using Graphpad Prism 6 and R version 3.4.0 together with RStudio version 1.0.143.

### Reporting summary

Further information on research design is available in the Nature Research Reporting Summary linked to this article.

## Supplementary Material

Reporting Summary

Source Data Fig 2

Source Data Fig 3

Source Data Fig 4

Source Data Fig 5

Source Data Fig1

Source Data Supp Fig 1

Source Data Supp Fig 2

Source Data Supp Fig 3

Supplementary information

Supplementary Video 1

Supplementary Video 2

Supplementary Video 3

Supplementary Video 4

Supplementary Video 5

Supplementary Video 6

Supplementary Video 7

Supplementary Video 8

Supplementary Video 9

Supplementary Video 10

Supplementary Video 11

Supplementary Video 12

## Data Availability

Raw data are available from the corresponding authors upon request. Source data are provided with this paper.

## One sentence summary

Molecular motors destroy microtubule lattice as they walk on it but a self-healing process incorporates new dimers in the damaged regions and prevents microtubule disassembly.
