# Supplementary material for: Self-repair protects microtubules from their destruction by molecular motors
Source: Nat Mater. Author manuscript; Available in PMC 2021 Dec 1. (PMC7611741; doi:10.1038/s41563-020-00905-0)
Supplement: Supplementary information [file EMS135429-supplement-Supplementary_information.pdf]

natureresearch

https://doi.org/10.1038/s41563-020-00905-0

Supplementary information

Self-repair protects microtubules from destruction by molecular motors

In the format provided by the authors and unedited

Self-repair protects microtubules from their destruction by molecular motors

Sarah Triclin[1]*, Daisuke Inoue[1,2]*, Jérémie Gaillard[1], Zaw Min Htet[3], Morgan E. DeSantis[3], Didier Portran[5], Emmanuel Derivery[6], Charlotte Aumeier[7], Laura Schaedel[1], Karin John[8], Christophe Leterrier[9], Samara L. Reck-Peterson[3,4], Laurent Blanchoin[1,10$], Manuel Théry[1,10$]

*these authors contributed equally to this work.

$ Address correspondence to: laurent.blanchoin@cea.fr or manuel.thery@cea.fr

1-University of Grenoble-Alpes, CEA, CNRS, INRA, Biosciences & Biotechnology Institute of Grenoble, UMR5168, LPCV, CytoMorpho Lab, 38054 Grenoble, France.

2-Department of Human Science, Faculty of Design, Kyushu University, 815-8540, Fukuoka, Japan.

3-Deptartment of Cellular and Molecular Medicine, and Cell and Developmental Biology Section, Division of Biological Sciences, University of California San Diego, 9500 Gilman Drive, La Jolla, CA 92093, USA

4-Howard Hughes Medical Institute, Chevy Chase MD, USA

5-CRBM - CNRS UMR 5237, Route de Mende, 34293 Montpellier Cedex 05, FRANCE

6-MRC Laboratory of Molecular Biology, Francis Crick Avenue, Cambridge Biomedical Campus, Cambridge CB2 0QH, UK

7-University of Geneva Department of Biochemistry, Science II 30, Quai Ernest-Ansermet
1205 Genève, Switzerland

8- University of Grenoble-Alpes, CNRS, Laboratoire Interdisciplinaire de Physique, 38000 Grenoble, France.

9- University of Aix Marseille, CNRS, INP UMR7051, Marseille, France

10- University of Paris, INSERM, CEA, Institut de Recherche Saint Louis, U976, HIPI, CytoMorpho Lab, 75010 Paris, France.

Supplementary information

Supplementary Methods
Supplementary References
Supplementary Figure legends
Supplementary Tables
Supplementary Movie legends

Supplementary Methods

Purification and labelling of brain tubulin

Bovine brain tubulin was purified in BRB80 buffer (80 mM 1,4-piperazinediethanesulfonic acid, pH 6.8, 1 mM ethylene glycol tetraacetic acid, and 1 mM MgCl2) according to previously published method[1]. Tubulin was purified from fresh bovine brain by three cycles of temperature-dependent assembly and disassembly in Brinkley Buffer 80 (BRB80 buffer; 80 mM PIPES, pH 6.8, 1 mM EGTA, and 1 mM MgCl2 plus 1 mM GTP)[2]. MAP-free neurotubulin was purified by cation-exchange chromatography (EMD SO, 650 M, Merck) in 50 mM PIPES, pH 6.8, supplemented with 1 mM MgCl2, and 1 mM EGTA (Malekzadeh-Hemmad et al., 1993). Purified tubulin was obtained after a cycle of polymerization and depolymerization.

Fluorescent tubulin (ATTO 488–labeled tubulin and ATTO 565–labeled tubulin) and biotinylated tubulin were prepared according to previously published method[3]. Microtubules from neurotubulin were polymerized at 37°C for 30 min and layered onto cushions of 0.1 M NaHEPES, pH 8.6, 1 mM MgCl2, 1 mM EGTA, 60% v/v glycerol, and sedimented by high centrifugation at 30°C. Then microtubules were resuspended in 0.1 M NaHEPES, pH 8.6, 1 mM MgCl2, 1 mM EGTA, 40% v/v glycerol and labeled by adding 1/10 volume 100 mM NHS-ATTO (ATTO Tec), or NHSBiotin (Pierce) for 10 min at 37°C. The labeling reaction was stopped using 2 volumes of 2X BRB80, containing 100 mM potassium glutamate and 40% v/v glycerol, and then microtubules were sedimented onto cushions of BRB80 supplemented with 60% glycerol. Our labelling ratio is: for tubulin 488 nm 1.6 to 1.8 fluorophore by dimers and for tubulin 565 nm 0.6 to 0.7 fluorophore by dimers. We used NanoDrop one UV-Vis spectrophotometer (Thermofisher) to determine the labelling efficiency. Microtubules were resuspended in cold BRB80. Microtubules were then depolymerised and a second cycle of polymerization and depolymerization was performed before use.

Purifications of molecular motors

Recombinant, truncated kinesin-1 motor protein was purified using bacterial expression as previously described[4]. Plasmid for the kinesin construct, pET17_K560_GFP_His, was purchased from Addgene (15219, Cambridge, MA). Plasmid for the kinesin construct (GFP and SNAP) gratefully provided by Dr. Ross. Recombinant, truncated kinesin-1 motor protein fusion proteins were transfected into Rosetta2 (DE3)-pLysS E. coli (VWR) and expression was induced with 0.2 mM IPTG for 16 h at 20°C. Harvested cells were resuspended in buffer A (50 mM sodium phosphate buffer pH 8, 250 mM NaCl) containing 0.1% Tween-20, 0.5 mM ATP and

protease inhibitors (Roche) and lysed by sonification. The lysates were centrifuged 30 min at 25000 g and 4°C. We add 20 mM Imidazole to the clear lysates and were loaded onto a NiSepharose HP column (GE Healthcare). The column was washed with buffer B (50 mM sodium phosphate buffer pH 6, 250 mM NaCl, 1 mM MgCl2, 0.1 mM ATP). Proteins were eluted in buffer C (50 mM sodium phosphate buffer pH 7.2, 250 mM NaCl, 500 mM Imidazole, 1 mM MgCl2, 0.1mM ATP) then desalted into PEM-100 buffer containing 0.1 mM ATP, and snap-frozen in liquid nitrogen.

Klp2 was purified according to previous publication[5]. Full-length Klp2 cDNA (gratefully provided by Dr. S Diez) was cloned into the pET-30a(+) expression vector (Novagen) with Snap Tag to N-Ter and His Tag to C-Ter. The recombinant N-terminal SNAP tagged and C-terminal His Tagged fusion proteins were transfected into Rosetta2 (DE3)-pLysS E. coli (VWR) and expression was induced with 0.1 mM IPTG for 16 h at 15°C. Harvested cells were resuspended in buffer A (50 mM sodium phosphate buffer pH 7.4, 2 mM MgCl2, 1 mM DTT, 0.1 mM ATP) containing 200 mM NaCl and protease inhibitors (Roche) and lysed in 0.05% v/v Triton X-100 (Sigma) by sonification. The lysates were centrifuged 30 min at 25000 g and 4°C. The clear lysates were loaded onto a NiSepharose HP column (GE Healthcare). The column was washed with buffer A containing 100 mM NaCl and 20 mM imidazole. Proteins were eluted in buffer A containing 100 mM NaCl and 250 mM imidazole then desalted into buffer A containing 100 mM NaCl without ATP. Gel filtration of Klp2 was performed on a fast protein liquid chromatography system with a HiLoad 16/600 Superdex 200 column (Amersham Pharmacia Biotech). Column equilibration and chromatography were performed in buffer A contining 100mM NaCl without ATP. Purified Klp2 was snap-frozen in liquid nitrogen. Snap-Klp2 was labelled with Snap Surface 488 (New England Biolabs) during purification.

Constitutive homodimer of Dyneins ($Dyn1_{331\ kDa}$) as previously described [6] were purified from yeast strain RPY1167 with the following genotype: MATa, his3-11,15, ura3-1, leu2-3,112, ade2-1, trp1-1, pep4Δ::HIS5, prb1Δ, PGAL1-ZZ-TEV-GFP-3XHA-GST- DYN1(331kDa)-gsDHA-KanR, pac1Δ, ndl1Δ::cgLEU2 according to [7].

Preparation of microtubules for gliding assay experiment.

Taxol stabilized microtubules were obtained by polymerising at 37°C a mixture of 20% labelled tubulin and unlabelled tubulin at 55 µM in BRB80 supplemented with 1 mM GTP, 5% DMSO and 2 mM MgCl2. The microtubule solution was then diluted with taxol buffer (80 mM Pipes, 1 mM EGTA, 1 mM MgCl2, 10 µM paclitaxel and ~1% DMSO; pH 6.8).

GMPCPP microtubules were elongated by stepwise addition of tubulin (20 % labelled) into microtubule seeds in the presence of 0.5 mM GMPCPP in 1x BRB80 until the tubulin concentration reaches 10 µM. Prepared GMPCPP microtubules were diluted in HKEM (10 mM Hepes pH7.2; 5 mM $MgCl_2$; 1 mM EGTA; 50 mM KCl).

Capped GDP microtubules were prepared by elongating microtubules from green-labelled GMPCPP seeds with 20 μM of red-labelled tubulin in HKEM supplemented with 0.5 mM GTP for 20 min at 37°C. These microtubules were then capped by being diluted in 5 μM of green-labelled tubulin in HKEM supplemented with 0.5 mM GMPCPP and incubation for 15 min at 37°C. Noteworthy, the preexisting red-tubulin is thus diluted in green-tubulin in the presence of GMPCPP. This led to the assembly of a second population of short microtubules that were less bright in the red channel than the capped GDP-microtubules.

Cover-glass micropatterning

The micropatterning technique was adapted from according to previous publication[5]. Cover glasses were cleaned by successive chemical treatments: 30 min in acetone with sonication, 15 min in ethanol (96%), rinsing in ultrapure water, 2 h in Hellmanex III (2% in water), and rinsing in ultrapure water. Cover glasses were dried using nitrogen gas flow, oxidized with oxygen plasma (5 min, 80 W, FEMTO; Diener Electronics) and incubated for one day in a solution of silane-PEG (30 kDa, PSB-2014, creative PEG works) 1 mg/ml in ethanol 96% and 0.1% of HCl, with gentle agitation at room temperature. Cover glasses were then successively washed in ethanol 96 % and ultrapure water, dried with nitrogen gas, and stored at 4°C. Passivated cover glasses were placed into contact with a photomask (Toppan) with a custom-made vacuum-compatible holder and exposed to deep UV (7 mW/cm 2 at 184 nm, Jelight) for 1 min. Deep UV exposure through the transparent micropatterns on the photomask created oxidized micropatterned regions on the PEG-coated cover glasses.

Microfluidic circuit fabrication and flow control

The microfluidic device was fabricated in PDMS (Sylgard 184, Dow Corning) using standard photolithography and soft lithography. The master mold was fabricated by patterning 50-μm thick negative photoresist (SU8 3050, Microchem, MA) by photolithography. A positive replica was fabricated by replica molding PDMS against the master. Prior to molding, the master mold was silanized (trichloro(1H,1H,2H,2H-perfluorooctyl)silane, Sigma) for easier lift-off. One inlet and one outlet ports were made in the PDMS device using 0.5 mm soft substrate punches (UniCore 0.5, Ted Pella, Redding, CA). Connectors to support the tubing were made out of PDMS cubes (0.5 cm side length) with a 1.2 mm diameter through hole. The connectors were bonded to the chip ports using still liquid PDMS as glue, which was used to coat the interface between the chip and the connectors, and was then rapidly cured on a hotplate at 120°C. Teflon tubing (Tefzel, inner diameter: 0.03" outer

diameter: 1/16”, Upchurch Scientific) was inserted into the two ports serving as outlets. Tubing with 0.01” inner and 1/16” outer diameter was used to connect the inlets to a computer- controlled microfluidic pump (MFCS-4C, Fluigent, Villejuif, France). Flow inside the chip was controlled using the MFCS-Flex control software (Fluigent).

Statistical analysis of microtubule lifetimes

Statistical test were performed using the free software package R (http://www.R-project.org).

Analysis of survival curves in the absence of glycerol :

Survival curves were compared using a two-sample non-parametric log-rank test. To extract life-times we averaged survival curves over all experiments (simple averages) and fitted experimental survival curves to a theoretical model derived from a Weibull distribution

$$S(t) = 1 + \alpha\left[e^{-\left(\frac{t}{\tau}\right)^{k}} - 1\right], \quad (1)$$

where k denotes a scale factor, τ denotes a life-time, and α denotes the fraction of microtubules which will break as time $t \rightarrow \infty$ (see Supplementary Table 1 and Supplementary Figure 2A). A scale factor k≈1 denotes an exponential decay, whereas a scale factor k>1 denotes an aging process, where the microtubule failure increases over time.

Analysis of survival curves in the presence of 10% glycerol :

Since microtubules depolymerized slowly in the presence of glycerol (≤1 μm.min^{-1}) and to exclude any variation in microtubule survival times due to length variations in the microtubules (see Supplementary Tables S11-S14) we compared only microtubules of the same length, i.e. microtubules were sorted according to their length into bins with a width of 2 μm. Survival curves were then compared using a two-sample non-parametric log-rank test. To extract life-times of microtubules we averaged survival curves over all experiments (simple averages) for microtubules of comparable length L=10±1 μm and fitted these curves to a theoretical model derived from a Weibull distribution similar to Eq. (1) with a lag time t_0

$$S(t) = 1 + \alpha\left[e^{-\left(\frac{t-t_0}{\tau}\right)^{k}} - 1\right], \quad (2)$$

where k denotes a scale factor, τ denotes a life-time, and α denotes the fraction of microtubules which will break as time $t \rightarrow \infty$ (see Supplementary Tables 2 and 3 and Supplementary Figure 2B, C and D).

Statistical analysis of tubulin incorporation sites

Incorporation of free tubulin into end-stabilized microtubules was characterized by analysing distances between incorporation sites and the length of incorporation spots. Only incorporation spots which were more than 3 μm apart (on the same microtubule) were counted as separate incorporation spots. To determine distances, microtubules were concatenated in a random order but such that microtubules containing incorporation spots alternated with microtubules containing no incorporation spots in a regular order. We then measured center-to-center distances between incorporation sites. The samples of distance distributions and length distributions were compared using a two–sample non-parametric Wilcoxon test.

Supplementary References

1. Vantard, M., Peter, C., Fellous, A., Schellenbaum, P. & Lambert, A. M. Characterization of a 100-kDa heat-stable microtubule-associated protein from higher plants. *Fed. Eur. Biochem. Soc. J.* **220**, 847–853 (1994).
2. Shelanski, M. Chemistry of the filaments and tubules of brain. *J. Histochem. Cytochamistry* **21**, 529–539 (1973).
3. Hyman, A. *et al.* Preparation of modified tubulins. *Methods Enzymol.* **196**, 478–85 (1991).
4. Pierce, D. W. & Vale, R. D. Assaying processive movement of kinesin by fluorescence microscopy. *Methods Enzymol.* **298**, 154–171 (1998).
5. Portran, D., Gaillard, J., Vantard, M. & Thery, M. Quantification of MAP and molecular motor activities on geometrically controlled microtubule networks. *Cytoskeleton* **70**, (2013).
6. Reck-Peterson, S. L. *et al.* Single-Molecule Analysis of Dynein Processivity and Stepping Behavior. *Cell* **126**, 335–348 (2006).
7. DeSantis, M. E. *et al.* Lis1 Has Two Opposing Modes of Regulating Cytoplasmic Dynein. *Cell* **170**, 1197-1208.e12 (2017).

Supplementary Figures

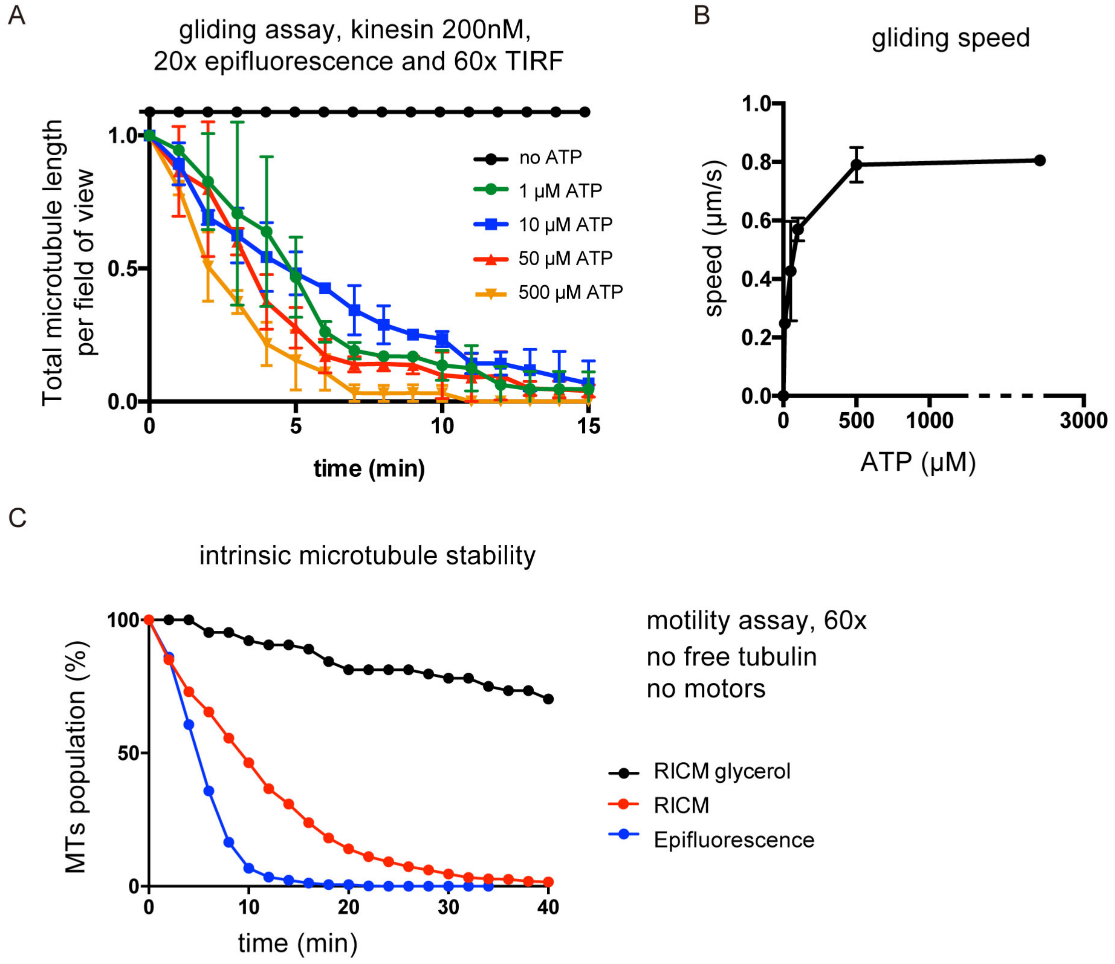

Supplementary Figure 1 – Role of ATP in microtubule destruction

A – Quantification of microtubule length variations at varied ATP concentrations ranging from 0 to 500 μM. The microtubule lengths were measured for all microtubules in the 600-μm-wide fields every minute during 15 minutes. Values of microtubule length were averaged and normalized with respect to the initial length (Number of independent experiments N=2, n=20). Error bars correspond to standard deviation.

B – Dependence of the velocity of kinesin-driven microtubules on ATP concentration ranging from 0 to 2.7 mM. Microtubules were stabilized by taxol. (Number of independent experiments N=2, n=20). Error bars correspond to standard deviation.

C - Survival curve of labelled microtubules under TIRF visualisation (blue curve), unlabelled microtubule under RICM visualisation (red curve) and unlabelled microtubule stabilized by 10% glycerol visualised with RICM (black curve). Control epifluorescence: n=659, control RICM: n=541, control RICM 10% glycerol: n=64.

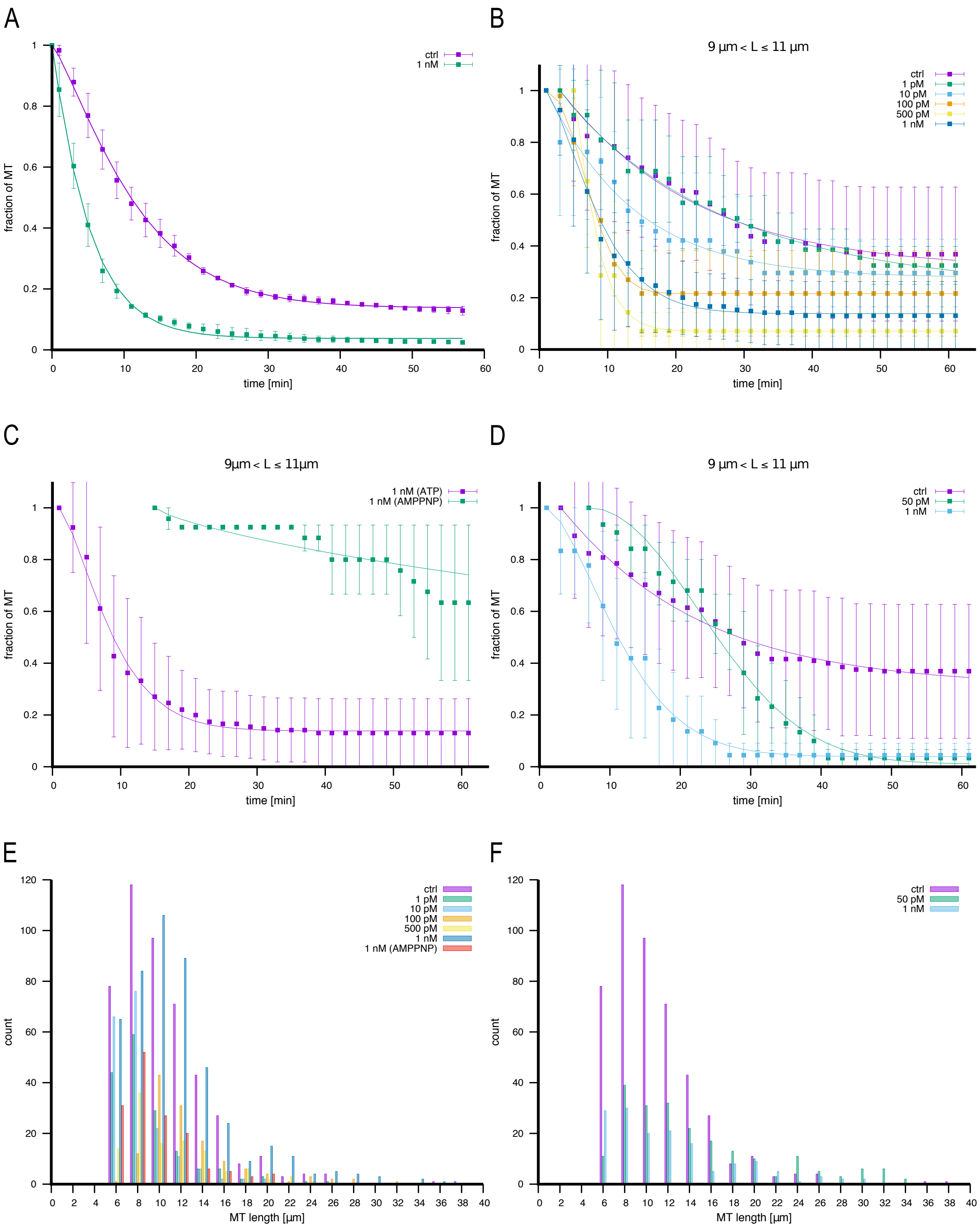

Supplementary Figure 2 – Statistical tests for the role of molecular motors

A-D – Experimental microtubule survival curves (symbols) and analytical curves (solid lines) obtained by a best fit to Eqs. (1) (A) or (2) (B, D) in the supplemental material. The experimental curves were obtained by averaging the proportion of MTs with respect to the initial amount of MTs (normal averages) over several experiments. The error bars represent the standard deviation. The results of the parameter fits for A, B and D are summarized in Supplementary Tables 1, 2 and 10. Differences

between survival curves where tested via a non-parametric two-sample log rank test. The p-value in (A) is <0.0001. The remaining p-values are summarized in Supplementary Tables 3-6 and 11.

A – Survival curves in the absence and presence of klp2 motors (1 nM) in the absence of glycerol.

B – Survivial curves of microtubules of length $L=10\pm1$ in the presence of klp2 motors with the concentrations as indiciated in the legend and in the presence of 10% glycerol.

C – Survivial curves of microtubules of length $L=10\pm1$ in the presence of klp2 motors (1 nM) with either ATP or the non-hydrolysable analogue AMPPNP and in the presence of 10% glycerol. The sample with 1 nM klp2 and ATP is the same as in (B).

D – Survivial curves of microtubules of length $L=10\pm1$ in the presence of dynein motors with the concentrations as indiciated in the legend and in the presence of 10% glycerol. The control sample is the same as in (B).

E,F – Histogramm of microtubule length distributions in survival experiments in the presence of klp2 (E) and dynein motors (F). Microtubule length distributions significantly vary between experimental conditions (see Supplementary Tables 8-9 and 13-14).

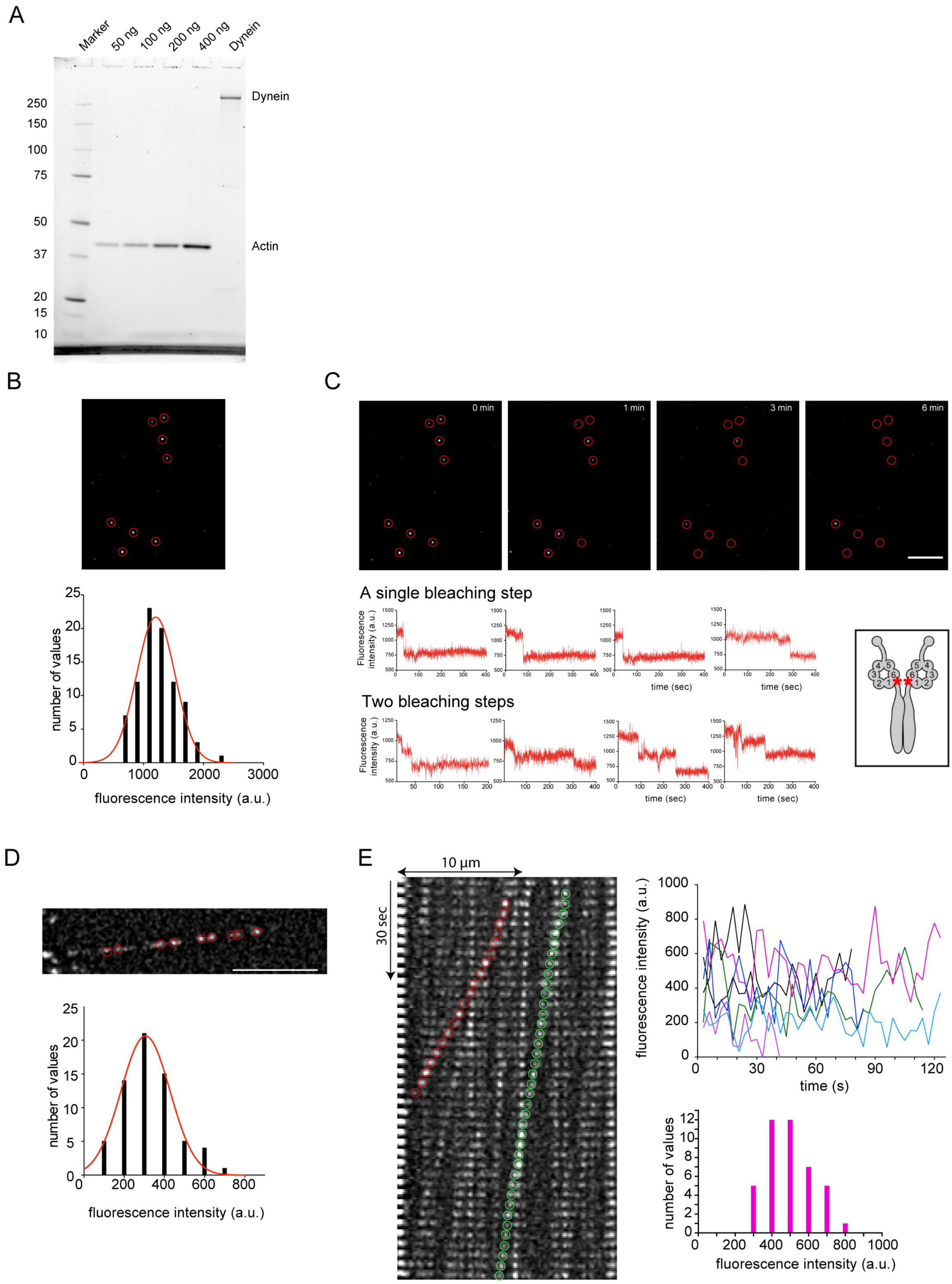

Supplementary Figure 3 – Experimental evidence for the use of single dyneins.

A - Sypro Red-stained SDS-PAGE gel of dynein purified from strain RPY1167 used in this work. Actin is used as a standard to determine protein amount. The right lane is representative of all dynein preps used in this study. The faint lower molecule weight band in the dynein prep is TEV protease left that was used to cleave dynein from

beads. This experiment has been performed once for this purification but is performed systematicallly for each purification and display the same degree of purity. B – TAMRA-labelled dyneins plated at 50 pM on the surface of a coverslip and photobleached by a high laser intensity in TIRF microscopy. The buffer was identical to the buffer used during the motility assay in Figure 3: 50 mM KCl; 16 mM pipes; 5 mM MgCl2; 1 mM EGTA; 2,6 mM ATP; 1 mM GTP; 10 mM hepes, 20 mM DTT; 3 mg/ml glucose; 20 μg/ml catalase; 100 μg/ml glucose oxidase, 0.3% BSA. Scale Bar: 10 μm. Red circles illustrate the region of interest in which fluorescence intensity was measured. Graph shows the distribution of fluorescence intensities in the first image of the photobleaching sequence. The distribution is not larger than the average value, suggesting the absence of clusters with multiple dimers of dyneins.

C – Graphs show the fluorescence intensities of bleaching particles over time. 96% (83/86) of the particles displayed one or two bleaching steps, as it can be expected for single dyneins dimers with two fluorophores [7], as illustrated in the schematic drawing. Red circles illustrate the region of interest in which fluorescence intensity was measured. Scale Bar: 10 μm.

D- Same conditions and image sequence as in Figure 3C. TAMRA-labelled dyneins were plated at 50 pM onto capped microtubule. Red circles illustrate the region of interest in which fluorescence intensity was measured. Scale bar : 5 μm. Graph shows the distribution of fluorescence intensities in the first image of the sequence. Intensities were lower than in the bleaching sequence since the aim was to monitor motor motion and microtubule survival, but the relative width of the distribution with respect to the average value was similar to the one measured in the photobleaching sequence, showing that the apparent heterogeneity of signal intensity was consistent with the presence single dynein dimers observed in the photobleaching experiments in panel B.

E- Same experiment as in D. The fuorescence intensities of particles were monitored over time as illustrated on the kymograph by colored circles and plotted on the top graph. The fluorescence intensity of isolated particles appear to fluctuate about 100% over time. The amplitude of the variations, represented in the bottom histogram, was similar to the amplitude measured over various particles in the first image (analysed in panel D). This showed that the origin of fluorescence variations were associated to the biochemical conditions and imaging procedure rather than to the presence of clusters of dimers of dynein.

A

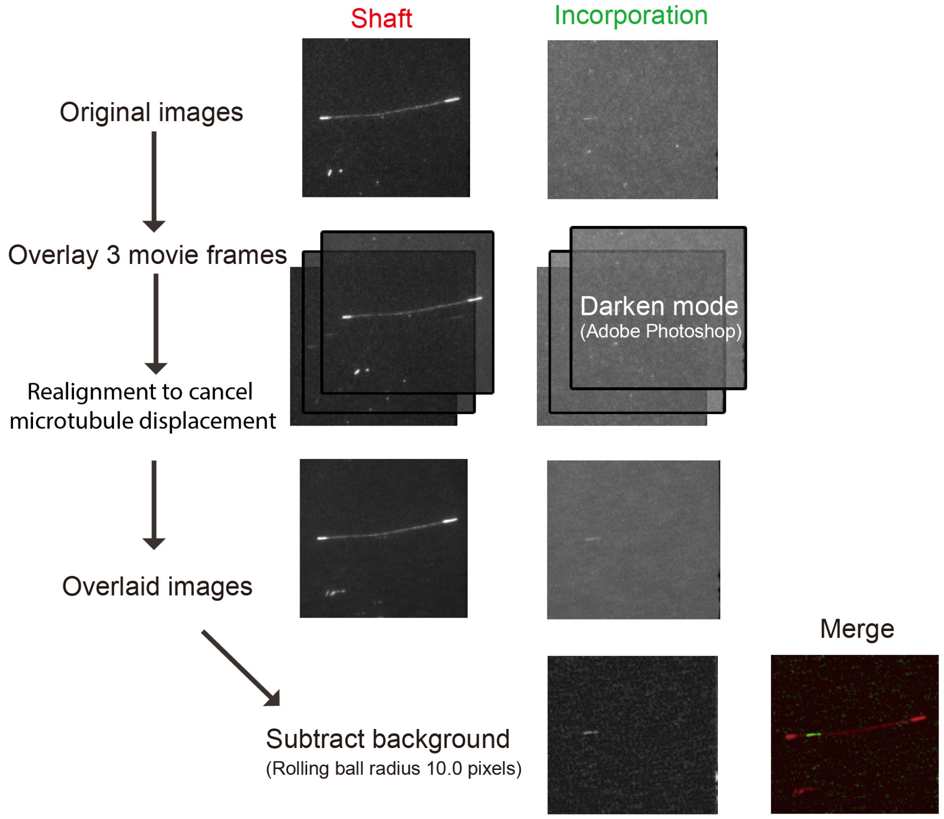
Shaft
Incorporation
Original images
Overlay 3 movie frames
Darken mode
(Adobe Photoshop)
Realignment to cancel
microtubule displacement
Overlaid images
Merge
Subtract background
(Rolling ball radius 10.0 pixels)

B

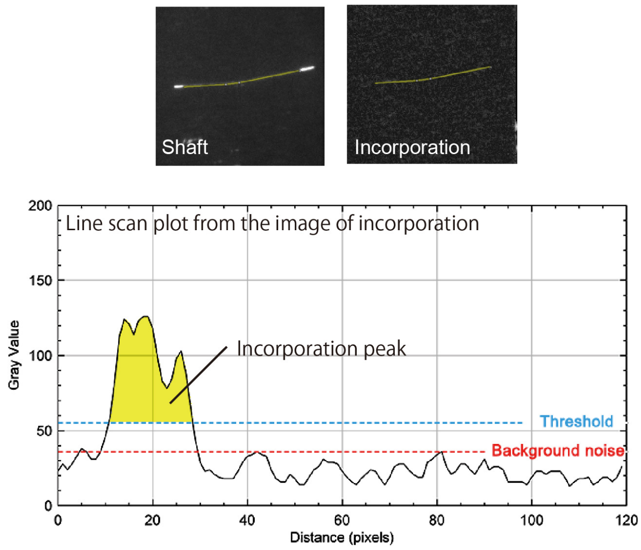
Shaft
Incorporation
Line scan plot from the image of incorporation
Incorporation peak
Threshold
Background noise
Gray Value
Distance (pixels)
200
150
100
50
0
0
20
40
60
80
100
120

Supplementary Figure 4 – image processing to detect incorporations

Movies were recorded by taking images every second during 30 s. Quantification of the incorporation of free green tubulin dimers in red microtubule shaft was done by measuring green fluorescence intensity along the microtubule using a line scan after image processing.

A- Three sequential frames were selected at an abitrarily given time point. Overlaid images were prepared by using Adobe Photoshop original macro for each channel by cancelling the displacement of microtubules. To overlay incorporation images, Photoshop layer mode “darken mode” was applied to each image in order to reduce background noise. The background of the overlaid image of incorporation was subtracted by Image J (Rolling ball radius 10.0 pixels).

B- The incorporation peak in the Image J line scan plot was defined as peaks above the threshold value 1.5 times higher than background peaks.

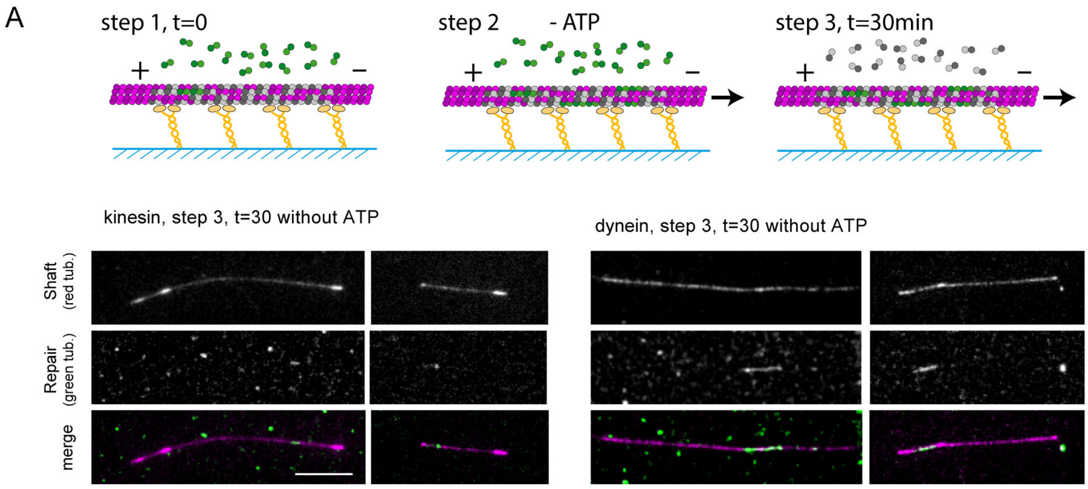

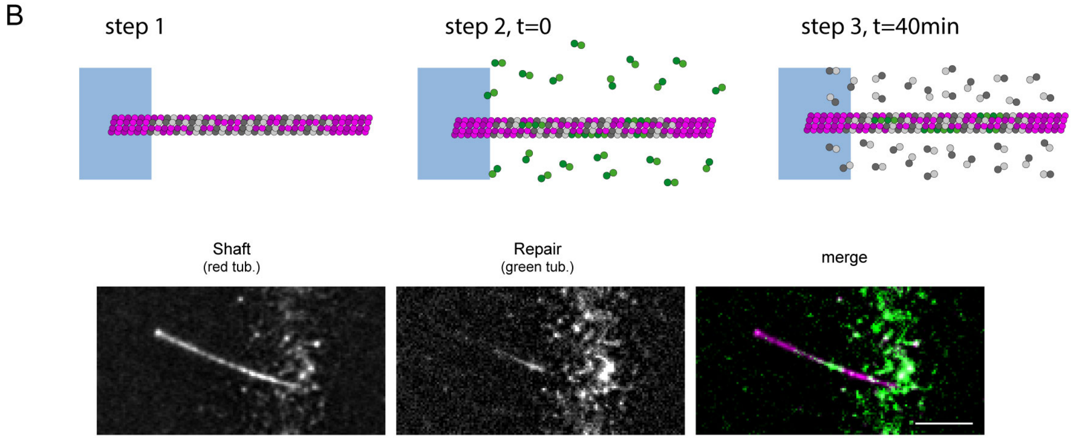

Supplementary Figure 5 – Spontaneous incorporation on microtubule lattice.

A- Images show the microtubule shaft (low intensity) with associated cap and seed (high intensity) (top row), the incorporation of free tubulin dimers (middle row), and the overlay of the two signals (bottom row) after 30 mn in absence of ATP, ie: no gliding condition. In these examples, microtubules are immobilized on kinesin-1 or dynein for 30 minutes. Scale bar: 10 μm.

B- Images show the microtubules shaft (left), the incorporation of free dimers tubulin (middle) and the overlay of the two signals (right) after 40 min of self-repair in control condition without molecular motors. Scale bar: 10 μm.

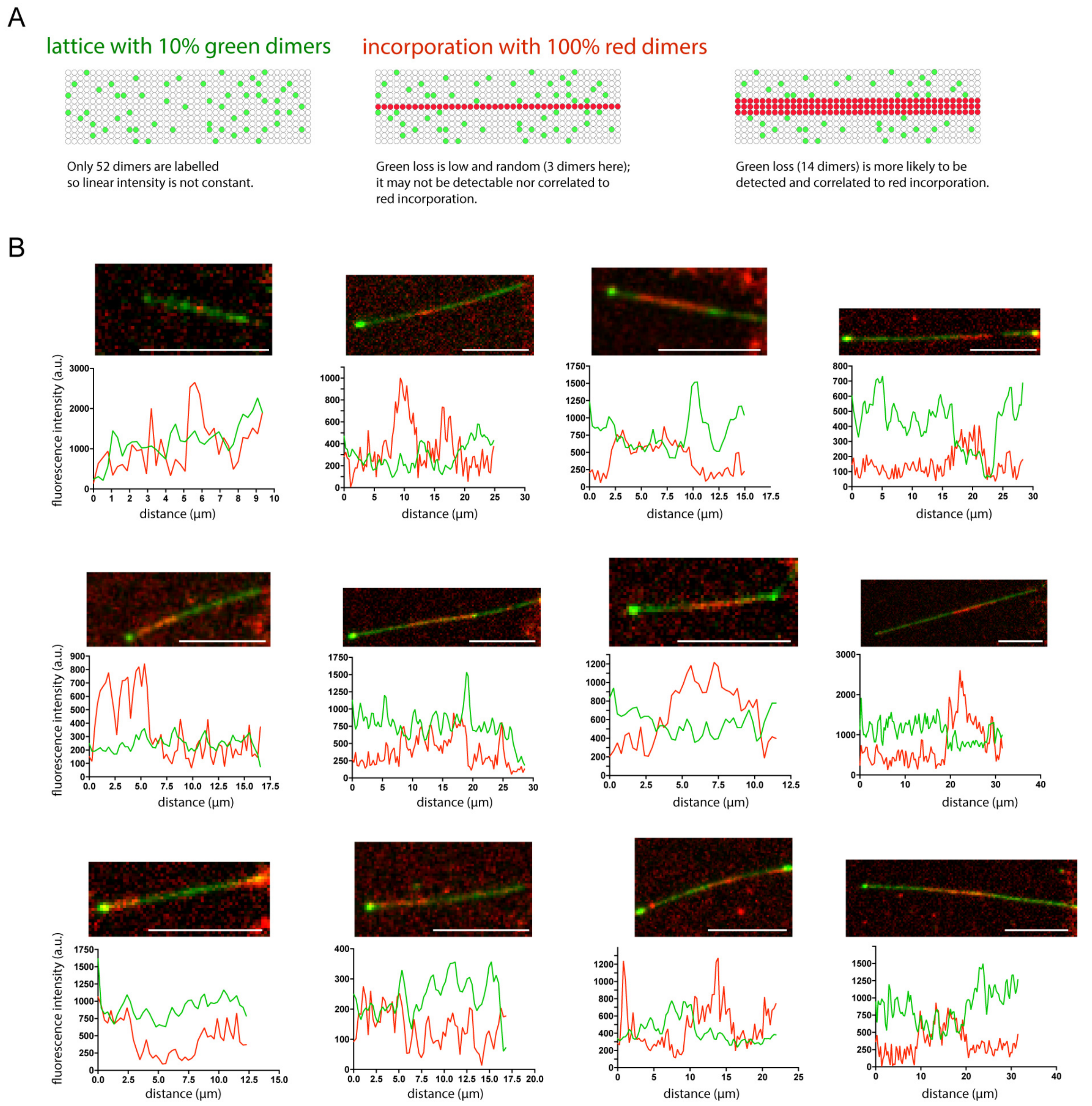

Supplementary Figure 6 – Replacement of green by red dimers at repair sites

A- Schematic representation of the repair process. Considering that all dimers are not labelled in the lattice, the green fluorescence intensity along the lattice is heterogeneous. So the loss of a protofilament may be associated to a low reduction of fluorescence which may not be detected. In addition, when detectable, the decrease of fluorescence is a probabilistic event which may not be correlated to the loss of material and thus to the gain of red fluorescence.

B- Examples of fluorescence linescan in green (initial lattice) and red (incorporation) fluorescences. Conditions are similar to Figure 5F. Examples on the left do not show a correlation whereas it is clearly visible in examples on the right.

Supplementary Tables

Microtubule life times in the presence of KINESINS without glycerol :

kpl2 conc.	amplitude α	scale factor k	life time τ [min]
Ctrl	0.862±0.003	1.24±0.02	12.3±0.1
1 nM	0.962±0.003	1.04±0.03	5.26±0.09

Supplementary Table 1 : Effect of klp2 motors on the failure of microtubules: Results of a parameter fit of a theoretical survival function derived from the Weibull distribution [Eq. (1), see Supplementary Methods] to the experimental survival curves of microtubuless (see Supplementary Figure 2A). The parameters are presented as mean±SD. A two-sample log-rank test gives a p-value of <0.0001. The total number of microtubules is N=588 (Ctrl) and N=1130 (1 nM klp2).

Microtubule lifetimes in the presence of KINESINS with 10% glycerol :

For the analysis, one experiment with control conditions was dropped, since during the whole observation period, only 1 out of 56 microtubules depolymerized.

kpl2 conc.	amplitude α	scale factor k	life time τ [min]	lag time t_0[min]
Ctrl	0.69±0.03	1.02±0.07	20±2	3
1 pM	0.78±0.03	0.96±0.06	25±2	3
10 pM	0.72±0.01	1.02±0.07	12.7±0.7	1
100 pM	0.784±0.002	2.10±0.06	7.47±0.08	1
500 pM	0.929±0.005	1.18±0.08	3.5±0.1	5
1 nM	0.862±0.005	1.39±0.06	8.7±0.2	1

Supplementary Table 2 : Effect of klp2 motors on the failure of microtubules in the presence of glycerol: Results of a parameter fit of a theoretical survival function derived from the Weibull distribution to [Eq. (2), see Supplementary Methods] the experimental survival curves of microtubules of the length 9 μm < L ≤ 11 μm (see Supplementary Figure 2B). The lag time t_0 was determined as the latest time, where all observed microtubules are intact. The parameters are presented as mean±SD. The results of statistical tests and the sample sizes are summarized in Supplementary Tables 3 to 9.

P-values for the comparison of survival curves of microtubules in the presence of KINESINS (two-sample log-rank test) :

klp2 conc .	1 pM	10 pM	100 pM	500 pM	1 nM
Ctrl	n.s.	n.s.	n.s.	n.s.	** 0.0015
1 pM	—	n.s.	n.s.	n.s.	*** 0.0003
10 pM	—	—	n.s.	n.s.	** 0.0088
100 pM	—	—	—	n.s.	n.s.
500 pM	—	—	—	—	n.s

Supplementary Table 3 : p-values of a two-sample log-rank test performed for microtubules of length 7 μm <L ≤9 μm in the presence of klp2 motors of varying concentration with 10% glycerol. The sample sizes are given in Supplementary Table 7.

kpl2 conc.	1 pM	10 pM	100 pM	500 pM	1 nM
Ctrl	n.s.	n.s.	**** <0.0001	**** <0.0001	**** <0.0001
1 pM	—	n.s.	** 0.0051	**** <0.0001	** 0.0047
10 pM	—	—	n.s.	** 0.0093	n.s.
100 pM	—	—	—	n.s.	n.s.
500 pM	—	—	—	—	n.s

Supplementary Table 4 : p-values of a two-sample log-rank test performed for microtubules of length 9 μm <L ≤11 μm in the presence of klp2 motors of varying

concentration with 10% glycerol. The sample sizes are given in Supplementary Table 7.

kpl2 conc.	1 pM	10 pM	100 pM	500 pM	1 nM
Ctrl	n.s.	n.s.	****<br><0.0001	n.s.	****<br><0.0001
1 pM	—	n.s.	**<br>0.0049	n.s.	n.s.
10 pM	—	—	*<br>0.0106	n.s.	n.s.
100 pM	—	—	—	n.s.	n.s.
500 pM	—	—	—	—	n.s

Supplementary Table 5 : p-values of a two-sample log-rank test performed for microtubules of length 11 μm <L ≤13 μm in the presence of klp2 motors of varying concentration with 10% glycerol. The sample sizes are given in Supplementary Table 7.

MT length	7 μm < L ≤ 9 μm	9 μm < L ≤ 11 μm	11 μm < L ≤ 13 μm
p-value	<0.0001	<0.0001	<0.0001

Supplementary Table 6 : p-values of a two-sampe log-rank test performed for microtubules of length as indicated in the table header for microtubule survival statistics in the presence of 1 nM klp2 motors either with ATP or AMPPNP . The sample sizes are given in Supplementary Table 7.

Summary of sample sizes of microtubule failure experiments in the presence of KINESINS and 10% glycerol:

MT length	$7\ \mu m < L \le 9\ \mu m$		$9\ \mu m < L \le 11\ \mu m$		$11\ \mu m < L \le 13\ \mu m$	
klp2 conc.	N_{total}	N_{obs}	N_{total}	N_{obs}	N_{total}	N_{obs}
Ctrl	118	60	97	63	71	52
1 pM	59	27	29	20	13	12
10 pM	76	35	22	16	11	9
100 pM	12	6	43	35	31	30
500 pM	36	21	16	15	17	13
1 nM	84	61	106	89	89	79
1 nM (AMPPNP)	52	24	27	9	20	10

Supplementary Table 7 : Summary of sample sizes for survival experiments of microtubules in the prescence of klp2 motors. Shown is the total number of microtubules in the sample (N_{total}) and the number of microtubules, which completely depolymerized during the observation period of ~60 min (N_{obs}). L denotes the approximate length of the microtubules. The condition 1 nM (AMPPNP) indicates microtubule failure in the presence of 1 nM klp2 motors and the non-hydrolysable analogue AMPPNP, instead of ATP.

Summary of the analysis of microtubule length distributions in the presence of KINESINS (normality Test, two-sample Wilcoxon test)

klp2 conc.	Mean [μm]	SD [μm]	p-value	N
Ctrl	11	4	<0.0001	466
1 pM	9	4	<0.0001	164
10 pM	8	3	<0.0001	187
100 pM	13	5	<0.0001	132
500 pM	11	4	<0.0001	113
1 nM	12	5	<0.0001	468
1 nM (AMPPNP)	10	3	<0.0001	148

Supplementary Table 8 : Mean±standard deviation (SD) of microtubule length distributions in microtubule failure experiments in the presence of kl2 motors and

10% glycerol. The p-value was obtained using a normality test (Shapiro-Wilk test). A p-value of <0.0001 indicates a non-Gaussian distribution. N denotes the sample size.

klp2 conc.	1 pM	10 pM	100 pM	500 pM	1 nM	1 nM AMPPNP
Ctrl	<0.0001	<0.0001	<0.0001	n.s.	0.0031	—
1 pM	—	0.0087	<0.0001	0.0001	<0.0001	—
10 pM	—	—	<0.0001	<0.0001	<0.0001	—
100 pM	—	—	—	<0.0001	0.0003	—
500 pM	—	—	—	—	n.s.	—
1 nM	—	—	—	—	—	<0.0001

Supplementary Table 9 : P-values of a non-parametric two-sample Wilcoxon test of the length distributions of microtubules (two-sided, unpaired) for microtubule failure experiments in the presence of klp2 motors and 10% glycerol. A p-value <0.0001 indicates a true-location shift in the distributions of the two samples. The sample sizes are given in Supplementary Table 7.

Microtubule lifetimes in the presence of DYNEIN motors with 10% glycerol :

For the analysis, one experiment with control conditions was dropped, since during the whole observation period, only 1 out of 56 microtubules depolymerized. The control conditions are the same as for klp2 motors.

dynein conc.	amplitude α	scale factor k	life time τ [min]	lag time [min]
Ctrl	0.69±0.03	1.02±0.07	20±2	3
50 pM	0.99±0.02	2.1±0.1	21.6±0.6	7
1 nM	0.961±0.008	1.55±0.08	12.4±0.3	1

Supplementary Table 10 : Effect of dynein motors on the failure of microtubules: Results of a parameter fit of a theoretical survival function derived from the Weibull distribution [Eq. (2), see Supplementary Methods] to the experimental survival curves of microtubules of the length 9 μm < L ≤ 11 μm (see Supplementary Fig. S2D). The lag time t_0 was determined as the latest time, where all observed microtubules are intact. The parameters are presented as mean±SD. The results of statistical tests and the sample sizes are summarized in Supplementary Tables 11 to 14.

P-values for the comparison of survival curves of microtubules in the presence of DYNEINS (two-sample log-rank test) :

MT length	7 μm < L ≤ 9 μm		9 μm < L ≤ 11 μm		11 μm < L ≤ 13 μm	
dynein conc.	50 pM	1 nM	50 pM	1 nM	50 pM	1 nM
Ctrl	****	****	*	****	n.s.	****
	<0.0001	<0.0001	0.0124	<0.0001		<0.0001
50 pM	—	n.s.	—	***	—	****
				0.0009		<0.0001

Supplementary Table 11 : p-values of a two-sample log-rank test performed for microtubules of length L as indicated in the header for microtubule survival statistics in the presence and absence of dynein motors. The control sampe (Ctrl) is the same as in Supplementary Table 7. The sample sizes are given in Supplementary Table 12.

Summary of sample sizes of microtubule failure experiments in the presence of DYNEINS and 10% glycerol:

MT length	7 μm < L ≤ 9 μm		9 μm < L ≤ 11 μm		11 μm < L ≤ 13 μm	
dynein conc.	N_{total}	N_{obs}	N_{total}	N_{obs}	N_{total}	N_{obs}
Ctrl	118	60	97	63	71	52
50 pM	39	39	31	30	32	32
1 nM	30	26	20	19	21	21

Supplementary Table 12 : Summary of sample sizes for survival experiments of microtubules in the prescence of dynein motors. Shown is the total number of microtubules in the sample (N_{total}) and the number of microtubules, which completely depolymerized during the observation period of ~60 min (N_{obs}). L denotes the approximate length of the microtubules. The control (Ctrl) is the same Ctrl sample as in Supplementary Table 7.

Summary of the analysis of microtubule length distributions in the presence of DYNEINS (normality Test, two-sample Wilcoxon test)

dynein conc.	Mean [μm]	SD [μm]	p-value	N
Ctrl	11	4	<0.0001	466
50 pM	15	7	<0.0001	211
1 nM	12	6	<0.0001	151

Supplementary Table 13 : Mean±standard deviation (SD) of microtubule length distributions in microtubule failure experiments in the presence of dynein motors and 10% glycerol. The p-value was obtained using a normality test (Shapiro-Wilk test). A p-value of <0.0001 indicates a non-Gaussian distribution. N denotes the sample size.

dynein conc.	50 pM	1 nM
Ctrl	<0.0001	n.s
50 pM	—	<0.0001

Supplementary Table 14 : P-values of a non-parametric two-sample Wilcoxon test of the length distributions of microtubules (two-sided, unpaired) for microtubule failure

experiments in the presence of dynein motors and 10% glycerol. A p-value <0.0001 indicates a true-location shift in the distributions of the two samples. The sample sizes are given in Supplementary Table 12.

Supplementary Movies legends

Supplementary Movie 1 - Gliding assay of stabilized microtubules.
Taxol-treated (left) and GMPCPP-microtubules (right), labelled with 20% fluorescent tubulin, gliding on a layer of kinesin-1.
Images were taken with a 20x objective in epifluorescence microscopy.
Acquisition frame rate: 1 image every 5 seconds during 15 minutes. Display: 25 im/s.
Scale bar: 100 μm.

Supplementary Movie 2 - Gliding assay of non-stabilized capped-GDP microtubules.
Capped-GDP-microtubules, labelled with 20% fluorescent tubulin, gliding on a layer of kinesin-1.
Images were taken with a 20x objective in epifluorescence microscopy.
Acquisition frame rate: 1 image every 5 seconds during 15 minutes. Display: 25 im/s.
Scale bar: 100 μm.

Supplementary Movie 3 - Microtubule breakage during gliding.
Capped-GDP-microtubules, labelled with 20% fluorescent tubulin, gliding on a layer of kinesin-1. Microtubules were automatically detected and tracked in order to reposition the visualisation field so as to keep the moving microtubule at the center of the field.
Images were taken with a 63x objective in TIRF microscopy with 1.5x magnifier.
Acquisition frame rate: 1 image every 5 seconds during 15 minutes. Display: 5 im/s.
Scale bar: 20 μm.

Supplementary Movie 4 - Microtubule breakage upon kinesin motor walk.
Capped-GDP-microtubules, labelled with 20% fluorescent tubulin, were assembled from micropatterned seeds and served as tracks for the motility of kinesins (10nM Klp2).
Images were taken with a 63x objective in TIRF microscopy with 1.5x magnifier.
Acquisition frame rate: 1 image every 3 seconds during 3 minutes. Display: 5 im/s.
Scale bar: 10 μm.

Supplementary Movie 5 – Microtubule breakage versus uncapping upon dynein motor walk.
Capped-GDP-microtubules, labelled with 20% fluorescent tubulin, were assembled from micropatterned seeds and served as tracks for the motility of kinesins (1nM

Klp2).
Images were taken with a 63x objective in TIRF microscopy with 1.5x magnifier.
Acquisition frame rate: 1 image every 3 seconds during 3 minutes. Display: 3 im/s.
Scale bar: 10 μm.

Supplementary Movie 6 – Single dynein motors walking on a capped microtubule.
Capped-GDP-microtubules, labelled with 20% fluorescent tubulin, were assembled from micropatterned seeds and served as tracks for the motility of dyneins (50 pM).
Images were taken with a 63x objective in TIRF microscopy with 1.5x magnifier.
Acquisition frame rate: 1 image every 3 seconds during 2 minutes. Display: 10 im/s.
Scale bar: 10 μm.

Supplementary Movie 7 – Microtubule breakage upon dynein motor walk.
Capped-GDP-microtubules, labelled with 20% fluorescent tubulin, were assembled from micropatterned seeds and served as tracks for the motility of dyneins (50 pM).
Images were taken with a 63x objective in TIRF microscopy with 1.5x magnifier.
Acquisition frame rate: 1 image every 3 seconds during 3 minutes. Display: 5 im/s.
Scale bar: 10 μm.

Supplementary Movie 8 - Free tubulin dimers protect microtubules during gliding assay on kinesins.
Capped-GDP-microtubules, labelled with 20% fluorescent tubulin, gliding on a layer of kinesin-1 in the presence of unlabelled free tubulin dimers.
Images were taken with a 20x objective in epifluorescence microscopy.
Acquisition frame rate: 1 image every 5 seconds during 15 minutes. Display: 25 im/s.
Scale bar: 100 μm.

Supplementary Movie 9 - Free tubulin dimers protect microtubules during gliding assay on dyneins.
Capped-GDP-microtubules, labelled with 20% fluorescent tubulin, gliding on a layer of dyneins in the presence of unlabelled free tubulin dimers.
Images were taken with a 20x objective in epifluorescence microscopy.
Acquisition frame rate: 1 image every 5 seconds during 15 minutes. Display: 25 im/s.
Scale bar: 100 μm.

Supplementary Movie 10- Free tubulin dimers protect microtubules during motility assay.
Capped-GDP-microtubules, labelled with 20% fluorescent tubulin, were assembled from micropatterned seeds and served as tracks for the motility of kinesins (10 nM Klp2) in the absence (left) or presence (right) of free tubulin dimers (14 μM).

Images were taken with a 63x objective in TIRF microscopy microscopy with 1.5x magnifier.
Acquisition frame rate: 1 image every 2 minutes during 1 hour. Display: 5 im/s.
Scale bar: 10 μm.

Supplementary Movie 11 - Free tubulin dimers incorporate along microtubules during gliding assay.
Capped-GDP-microtubules, with the shaft labelled with 3% red fluorescent tubulin and the stable ends labelled with 20% red fluorescent tubulin (middle column), gliding on a layer of kinesins (kinesin-1) in the presence of unlabelled fluorescent free tubulin dimers after a 30-minute-long exposure to 14 μM free green fluorescent tubulin dimers (left column).
Images were taken with a 63x objective in TIRF microscopy with 1.5x magnifier.
Acquisition frame rate: 1 image every second during 30 seconds. Display: 5 im/s.
Scale bar: 20 μm.

Supplementary Movie 12 - Free tubulin dimers incorporate along microtubules during motility assay.
Capped-GDP-microtubules, labelled with 3% red fluorescent tubulin, were assembled from micropatterned seeds and served as tracks for the motility of kinesins (10 nM Klp2) in the presence of green free tubulin dimers (14 μM) which were further replaced by unlabelled dimers prior to imaging.
Images were taken with a 63x objective in TIRF microscopy with 1.5x magnifier.
Acquisition frame rate: 1 image every second during 30 seconds. Display: 5 im/s.
Scale bar: 10 μm.
